# Association of Trauma Center Designation With Postdischarge Survival Among Older Adults With Injuries

**DOI:** 10.1001/jamanetworkopen.2022.2448

**Published:** 2022-03-16

**Authors:** Molly P. Jarman, Ginger Jin, Joel S. Weissman, Arlene S. Ash, Jennifer Tjia, Ali Salim, Adil Haider, Zara Cooper

**Affiliations:** 1 Center for Surgery and Public Health, Department of Surgery, Brigham and Women’s Hospital, Boston, Massachusetts; 2 Department of Surgery, Harvard Medical School, Boston, Massachusetts; 3 Department of Population and Quantitative Health Sciences, University of Massachusetts Medical School, Worchester; 4 Division of Trauma, Burn and Surgical Critical Care, Department of Surgery, Brigham and Women’s Hospital, Boston, Massachusetts; 5 Medical College, The Aga Khan University, Karachi, Pakistan

## Abstract

**Importance:**

Trauma centers improve outcomes for young patients with serious injuries. However, most injury-related hospital admissions and deaths occur in older adults, and it is not clear whether trauma center care provides the same benefit in this population.

**Objective:**

To examine whether 30- and 365-day mortality of injured older adults is associated with the treating hospital’s trauma center level.

**Design, Setting, and Participants:**

This prospective, population-based cohort study used Medicare claims data from January 1, 2013, to December 31, 2016, for all fee-for-service Medicare beneficiaries 66 years or older with inpatient admission for traumatic injury in 2014 to 2015. Data analysis was performed from January 1 to June 31, 2021. Preinjury health was measured using 2013 claims, and outcomes were measured through 2016. The population was stratified by anatomical injury pattern. Propensity scores for level I trauma center treatment were estimated using the Abbreviated Injury Scale, age, and residential proximity to trauma center and then used to match beneficiaries from each trauma level (I, II, III, and IV/non-trauma centers) by injury type.

**Exposure:**

Admitting hospital’s trauma center level.

**Main Outcomes and Measures:**

Case fatality rates (CFRs) at 30 and 365 days after injury, estimated in the matched sample using multivariable, hierarchical logistic regression models.

**Results:**

A total of 433 169 Medicare beneficiaries (mean [SD] age, 82.9 [8.3] years; 68.4% female; 91.5% White) were included in the analysis. A total of 206 275 (47.6%) were admitted to non-trauma centers and 161 492 (37.3%) to level I or II trauma centers. Patients with isolated extremity fracture had the fewest deaths (365-day CFR ranged from 16.1% [95% CI, 11.2%-22.4%] to 17.4% [95% CI, 11.8%-24.6%] by trauma center status). Patients with both hip fracture and traumatic brain injury had the most deaths (365-day CFRs ranged from 33.4% [95% CI, 25.8%-42.1%] to 35.8% [95% CI, 28.9%-43.5%]).

**Conclusions and Relevance:**

These findings suggest that older adults do not benefit from existing trauma center care, which is designed with younger patients in mind. There is a critical need to improve trauma care practices to address common injury mechanisms and types of injury in older adults.

## Key Points

**Question:**

Is long-term mortality of injured older adults associated with the treating hospital’s trauma center level?

**Findings:**

In this population-based cohort study of long-term mortality among 433 169 older Medicare beneficiaries with injuries, mortality rates did not vary by trauma center status.

**Meaning:**

These findings suggest that older adults do not benefit from existing trauma center care in the same ways as younger adults, indicating a need for revised trauma care guidelines and clinical practices that meet the needs of injured older adults.

## Teaser

This cohort study assesses the association between mortality rates of older adults with injuries and the treating hospital’s trauma center level.

## Introduction

Nearly 3 000 000 US older adults experience traumatic injury each year, resulting in 50 000 deaths and $19 billion in lifetime health care costs for survivors.^1^ National guidelines recommend trauma center (TC) care for injured older adults when possible,^2^ based on evidence that younger, critically injured patients benefit from TC care, risk of death after injury increases with age, and emergency medical services (EMS) personnel may underestimate injury severity in older adults.^2^ Although the association between age and injury mortality is well documented,^3,4^ it remains unclear whether older adults benefit from TC care as much as younger patients, particularly in the context of injury from low-energy, blunt mechanisms most common among older adults.

Evidence supporting TC care for older adults is outdated or limited in terms of generalizability to the full range of patients covered by current guidelines. The National Study on the Costs and Outcomes of Trauma, the most rigorous study of TC effectiveness to date, demonstrated a 40% reduction in 1-year mortality for trauma patients younger than 55 treated at TCs and no difference in mortality between older adults treated at TCs and non-trauma centers (NTCs).^5^ Several subsequent studies^6,7,8^ examined TC effectiveness in the geriatric population. One study^6^ using the Florida trauma registry demonstrated reduced inpatient mortality for older adults treated at TCs but did not assess long-term outcomes. A study^7^ using the Oklahoma trauma registry found short-term mortality benefits of TC care for older adults but excluded hip fractures attributed to osteoporosis. In contrast, a study^8^ using trauma registry data on critically injured patients from Utah and Northern California suggested higher mortality for older adults treated at TCs. In 2017, the National Academies of Sciences, Engineering, and Medicine noted sparse evidence informing best practices for geriatric trauma care as a significant weakness of the US trauma care system.^9^

To improve our understanding of geriatric trauma outcomes and inform guidelines for clinical management of injured older adults, we examined a nationally representative sample of Medicare beneficiaries treated at TCs and NTCs, stratified by injury type. We hypothesized that 1-year mortality for older adults with isolated injuries typical of low-energy mechanisms would not be associated with TC designation.

## Methods

This study was reviewed by the Mass General Brigham Institutional Review Board and approved with a waiver of consent based on use of data with limited indirect identifiers. This study followed the Strengthening the Reporting of Observational Studies in Epidemiology (STROBE) reporting guideline.

### Population and Data Sources

**Figure 1.**
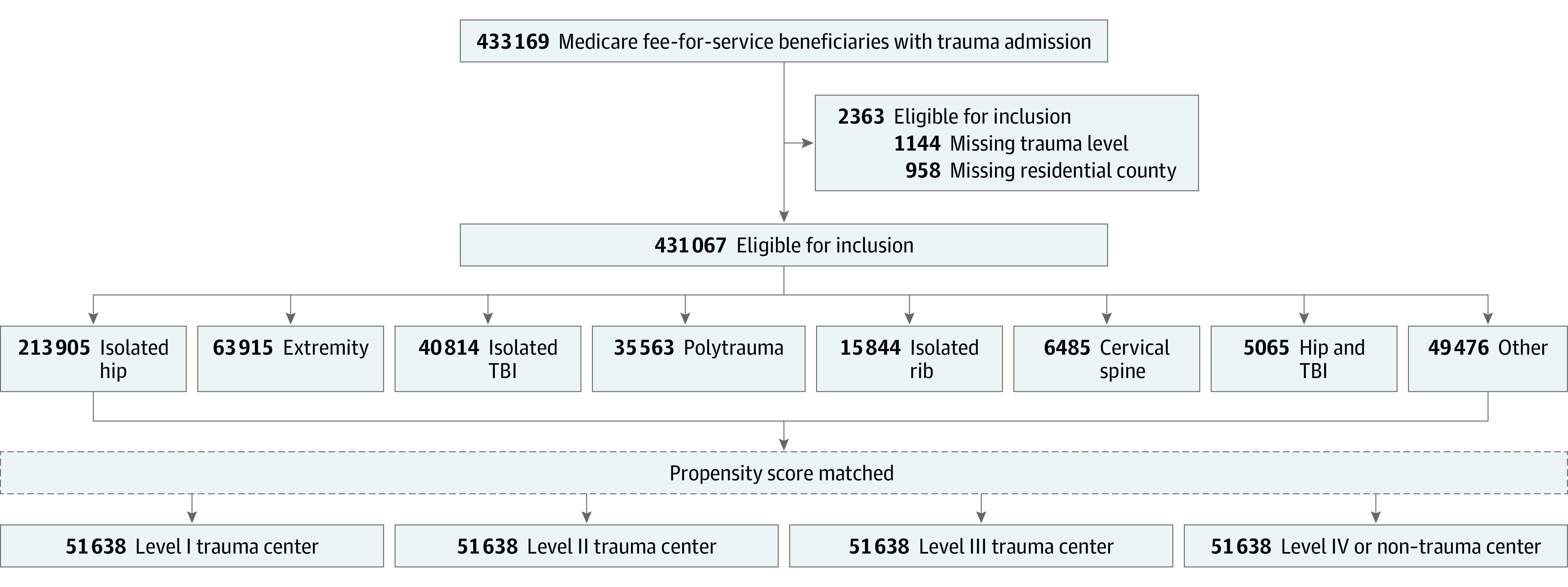
Flowchart of Inclusion and Exclusion Criteria Medicare beneficiaries were identified for inclusion based on injury diagnosis, excluded based on missing trauma center level or residential county, categorized based on injury type, and propensity score matched to create the final analytic cohort. TBI indicates traumatic brain injury.

Using Medicare claims from Inpatient and Outpatient Research Identifiable Files,^10,11^ we identified 433 169 fee-for-service beneficiaries aged 65 years or older diagnosed with traumatic injury between January 1, 2014, and December 31, 2015, resulting in inpatient admission (Figure 1). Traumatic injury was defined based on the 2015 National Trauma Data Standard,^12^ using *International Classification of Diseases, Ninth Revision *(*ICD-9*)* International Statistical Classification of Diseases and Related Health Problems, Tenth Revision *(*ICD-10*) (eTable 1 in the Supplement). We excluded patients who died in the emergency department because these early trauma deaths are likely attributable to nonsurvivable injuries.^13^ We also excluded patients with a primary noninjury diagnosis, with unknown county of residence, and patients treated at hospitals with unknown TC status because of missing or invalid facility identification numbers. Beneficiaries were included based on the first observed qualifying injury in the study period (ie, the index event). We used Medicare claims data from January 1, 2013, to December 31, 2014, to estimate preinjury health status and claims through December 31, 2016, to assess 365-day mortality.

We determined TC status by linking index injury encounters with data from the American Trauma Society Trauma Information Exchange Project (TIEP),^14^ using American Hospital Association identification numbers. Both American College of Surgeons TC verification and state designation were considered, with the most advanced level of care taking precedence in case of conflict (ie, if designated as American College of Surgeons level I and state level II, then coded as level I), consistent with previous research^15^ demonstrating similar outcomes between verification and designation types. Hospitals without TIEP records were considered NTCs. We used county of residence and TC addresses from TIEP to determine residential proximity to a TC. The TCs were attributed to the county where their coordinates mapped after geocoding with ArcGIS, version 10.7.1 (ESRI).

### Variables

Our primary outcomes of interest were mortality rates at 30 and 365 days after the index event. Mortality was determined based on date of death in the Medicare Master Beneficiary Summary File, which validates date of death for 99% of deceased beneficiaries.^16^

Our primary independent variable was level of TC verification and designation at the hospital where the beneficiary received definitive care. We considered each TC level separately (level I, level II, level III, and level IV/NTC). For beneficiaries with multiple encounters in a single day, we assumed patients moved to higher levels of care (ie, for a beneficiary with same-day NTC emergency department and level II TC inpatient encounters, a level II TC is the presumed definitive site).

Covariates in our analysis were Abbreviated Injury Scale^17^ (AIS) anatomical body region scores, injury type, age at time of injury, sex, race, Charlson Comorbidity Index (CCI) score,^18^ frailty, and residential proximity to a TC. The AIS anatomical scores (range, 0-6, with 0 indicating no injury and 6 indicating maximal injury severity) were determined by body region using a SAS macro adapted from ICD Programs for Injury Categorization.^19,20^ After review of the most common injury diagnosis patterns, injury type was categorized based on diagnosis codes and AIS scores as cervical spine fracture, hip fracture with traumatic brain injury (TBI), isolated hip fracture, isolated TBI, isolated rib fracture, other injury (ie, meets National Trauma Data Bank criteria, not otherwise categorized), other isolated extremity fracture, and multiple traumas (Table 1). Age was calculated based on dates of birth and index event. Sex (female/male) and race (Asian, Black, Hispanic, White, unknown, or other [including ≥2 races, known race not otherwise listed]) were used as coded in the Medicare Master Beneficiary Summary File. Using validated methods,^21^ we calculated CCI scores using all inpatient and outpatient diagnoses during the 365-day period that preceded the index event. Likewise, frailty was estimated using the validated Claims-Based Frailty Index^22^ and a 365-day look-back period, with frailty scores of 0.25 or less considered not frail and scores greater than 0.25 considered frail. County-level TC proximity, which has been reported to be associated with trauma mortality rates,^23^ was coded as TC in the county of residence, in an adjacent county, or no TC access.

**Table 1.** Injury Subtype Definitions

Injury subtype	Definition
Isolated hip fracture	Hip fracture diagnosis, no other *ICD* code for extremity injury, and AIS score <2 for body regions other than extremity
Other extremity fracture	Diagnosis code for any extremity fracture other than hip or femur and AIS score <2 for all body regions other than extremity
Isolated TBI	AIS score ≥2 for head and AIS score <2 for all body regions other than head
Isolated rib fracture	Diagnosis code for rib fracture and AIS score <2 for all body regions other than chest
Cervical spine fracture	Diagnosis code for cervical spine fracture and AIS score <2 for all body regions other than head/neck
Hip fracture and TBI	Hip fracture diagnosis, AIS≥2 for head, and AIS score <2 for body regions other than head/neck and extremity
Multiple traumas	AIS score ≥3 for ≥2 body regions and not otherwise categorized
Other injury	NTDB-eligible injury diagnosis and not otherwise categorized

Abbreviations: AIS, Abbreviated Injury Scale; *ICD, International Classification of Diseases*; NTDB, National Trauma Data Bank; TBI, traumatic brain injury.

### Statistical Analysis

Prehospital triage decisions made by EMS personnel determined assignment to TC and NTC treatment, influenced by patient-level injury, demographic characteristics, and geographic location of the injury incident. To minimize the impact of bias in treatment assignment on our findings, we stratified patients based on injury type and used propensity score matching to improve balance of key characteristics between the treatment groups. We estimated the likelihood of treatment at a level I center within each injury type stratum based on AIS anatomical scores, age, and TC proximity. Propensity scores were used in a 1:1:1:1 greedy, nearest neighbor match without replacement across all TC levels, within each injury type. Standardized mean differences were used to assess distribution of covariates across TC levels before and after matching.

The full population was used in bivariable logistic regression models, and the matched sample was used in hierarchical multivariable logistic regression models with hospital level to assess the association between TC level and injury mortality within each injury type. Unadjusted (unmatched, bivariable) and adjusted (matched, multivariable) odds of death were modeled for 30- and 365-day mortality. Multivariable logistic regression models were adjusted for factors that contribute to individual mortality risk (beneficiary sex, race, CCI score, and frailty) but not included in triage algorithms. Propensity score variables were included in multivariable regression models to account for residual imbalance between treatment groups. Regression models were then used to estimate crude (unadjusted) and adjusted marginal probabilities of death (interpreted as case fatality rates [CFRs]) for each injury type across TC levels. Models were not adjusted to account for multiple comparisons because this approach is more conservative in the context of our hypothesis. Data preparation and analysis used SAS software, version 9.4 (SAS Institute Inc). Statistical significance was determined based on an a priori threshold of α < .05.

## Results

This study assessed 433 169 Medicare fee-for-service beneficiaries (mean [SD] age, 82.9 [8.3] years; 68.4% female; 5860 [1.4%] Asian, 17 256 [4.0%] Black, 5233 [1.2%] Hispanic, 396 320 [91.5%] White, 1089 [0.3%] unknown, and 5309 [1.2%] other) with a primary diagnosis for traumatic injury that resulted in inpatient admission or emergency department observation between January 1, 2014, and December 31, 2015. We excluded 2102 patients (0.5%) because of missing TC designation (n = 1144 [0.3%]) and missing residential county (n = 958 [0.2%]). The composition of the cohort is illustrated in Figure 1.

Table 2 describes the demographic, health, and injury characteristics by TC level before and after matching. In the full population, patients treated at NTCs were more likely than those treated at TCs to be older than 85 years and to live in a county without a TC. Compared with patients at other trauma levels, patients at level I TCs were more likely to be Black (24 694 [36.7%]) and male (24 694 [36.7%]). Compared with patients at NTCs and level III TCs, patients at level I/II TCs were more likely to have a TBI (10 581 [15.7%] at level I TCs and 11 978 [12.7%] at level II TCs), cervical spine fracture (1664 [2.5%] at level I TCs and 2097 [2.2%] at level II TC), or multiple traumas (10 830 [16.1%] at level I TCs and 10 382 [11.0%] at level II TCs). Frailty and comorbidity scores did not vary substantially across TC levels. After propensity score matching, distributions of age, TC proximity, and injury characteristics were comparable across TC levels, and variations in sex and race across trauma levels were attenuated. Distributions of demographic, health, and injury characteristics by TC level for each injury subtype are available in eTables 2 to 9 in the Supplement, and standardized mean differences are illustrated in the eFigure in the Supplement.

**Table 2.** Population Characteristics by Trauma Level in Full Population and After Propensity Score Matching

Characteristic	No. (%)			Level III (n = 62 830)		Non-trauma center (n = 206 275)	
	Level I (n = 67 485)	Level II (n = 94 007)	SMD^a^	No. (%)	SMD^a^	No. (%)	SMD^a^
**Full population**							
Age group, y							
65-74	15 804 (23.4)	18 865 (20.1)	−0.12	12 144 (19.3)	−0.13	35 198 (17.0)	−0.06
75-84	23 218 (34.4)	32 638 (34.7)		22 228 (35.4)		70 002 (33.9)	
≥85	28 463 (42.2)	42 574 (45.3)		28 458 (45.3)		101 475 (49.1)	
CCI score							
0	21 320 (31.6)	30 066 (31.9)	0.04	19 525 (31.1)	0.03	66 675 (32.3)	0.02
1	16 072 (23.8)	22 749 (24.2)		15 282 (24.3)		50 377 (24.4)	
2	11 130 (16.5)	15 463 (16.4)		10 466 (16.7)		34 183 (16.5)	
≥3	18 963 (28.1)	25 799 (27.4)		17 557 (27.9)		55 440 (26.8)	
Frail	33 847 (50.2)	48 800 (51.9)	0.05	32 748 (52.1)	−0.06	110 001 (53.2)	−0.02
Sex							
Female	42 694 (63.3)	63 330 (67.3)	−0.12	44 207 (70.4)	−0.21	148 754 (71.9)	−0.07
Male	24 694 (36.7)	30 677 (32.7)		18 623 (29.6)		57 521 (28.1)	
Race							
Asian	1265 (1.9)	1082 (1.2)	0.08	404 (0.6)	0.16	3109 (1.5)	0.01
Black	4345 (6.4)	3142 (3.3)	0.20	1875 (2.9)	0.23	7894 (3.8)	0.04
Hispanic	766 (1.1)	952 (1.0)	0.02	758 (1.2)	−0.01	2757 (1.3)	−0.01
White	59 781 (88.6)	87 567 (93.1)	−0.22	58 894 (93.7)	−0.26	190 078 (91.9)	−0.04
Other^b^	1083 (1.6)	1104 (1.2)	0.05	761 (1.2)	0.05	2361 (1.1)	0.01
Unknown	245 (0.4)	230 (0.2)	0.03	138 (0.2)	0.04	476 (0.2)	0.01
Trauma center proximity							
In county	53 621 (79.5)	70 214 (74.6)	−0.17	46 424 (73.9)	−0.19	118 550 (57.4)	−0.17
Adjacent county	11 297 (16.7)	1872 (20.1)		12 870 (20.5)		67 008 (32.4)	
No trauma center	2567 (3.8)	4991 (21.8)		3536 (14.6)		21 117 (10.2)	
AIS score ≥3							
Head and neck	14 677 (21.8)	14 649 (15.6)	0.22	5118 (8.2)	0.59	14 218 (6.9)	0.17
Face	59 (0.1)	38 (0.1)	0.12	<10^c^	0.27	21 (0.1)	0.07
Chest	5926 (8.8)	5104 (5.4)	0.20	2566 (4.1)	0.34	5322 (2.6)	0.11
Abdomen and pelvis	1069 (1.6)	782 (0.8)	0.10	303 (0.5)	0.19	562 (0.3)	0.05
Extremity	25 614 (37.9)	41 384 (43.9)	−0.20	34 562 (55.0)	−0.55	112 604 (54.5)	−0.14
External	245 (0.4)	114 (0.1)	0.13	89 (0.1)	0.27	216 (0.1)	0.08
Injury subtype							
Cervical spine fracture	1664 (2.5)	2097 (2.2)	NA	733 (1.2)	NA	1991 (1.0)	NA
Hip fracture and TBI	830 (1.2)	1359 (1.4)	NA	754 (1.2)	NA	2122 (1.0)	NA
Isolated fracture							
Hip	23 827 (35.3)	40 363 (42.9)	NA	34 244 (54.5)	NA	115 471 (55.9)	NA
Rib	3058 (4.5)	3838 (4.1)	NA	2327 (3.7)	NA	6621 (3.2)	NA
Isolated TBI	10 581 (15.7)	11 978 (12.7)	NA	4575 (7.3)	NA	13 680 (6.6)	NA
Other extremity fracture^d^	8725 (12.9)	13 235 (14.1)	NA	9500 (15.1)	NA	32 455 (15.7)	NA
Other injury^e^	7970 (11.8)	10 825 (11.5)	NA	6575 (10.5)	NA	24 106 (11.7)	NA
Multiple traumas	10 830 (16.1)	10 382 (11.0)	NA	4122 (6.6)	NA	10 229 (5.0)	NA
**After propensity score matching**							
Total No.	51 638	51 638		51 638		51 638	
Age group, y							
65-74	10 561 (20.5)	10 562 (20.5)	0	10 532 (20.5)	0	10 565 (20.5)	0
75-84	17 561 (34.0)	17 569 (34.0)		17 608 (34.0)		17 546 (34.0)	
≥85	23 516 (45.5)	23 507 (45.5)		23 498 (45.5)		23 527 (45.5)	
CCI score							
0	16 496 (31.9)	16 647 (32.2)	0.07	15 965 (30.9)	0.02	16 798 (32.5)	0.08
1	12 091 (23.4)	12 414 (24.0)		12 572 (24.4)		12 614 (24.4)	
2	8497 (16.5)	8507 (16.5)		8646 (16.7)		8485 (16.4)	
≥3	14 554 (28.2)	8646 (27.3)		14 455 (27.9)		13 741 (26.6)	
Frail	26 311 (50.9)	26 579 (51.5)	−0.01	26 935 (52.2)	−0.03	27 429 (53.1)	−0.02
Sex							
Female	34 882 (67.6)	35 535 (68.8)	−0.01	36 069 (69.9)	−0.07	36 251 (70.2)	−0.03
Male	16 756 (32.4)	16 103 (31.2)		15 569 (30.1)		15 387 (29.8)	
Race							
Asian	897 (1.7)	592 (1.2)	0.01	322 (0.6)	0.14	960 (1.9)	0
Black	3217 (6.2)	1746 (3.4)	0.03	1585 (3.1)	0.21	2171 (4.2)	0.02
Hispanic	551 (1.1)	519 (1.0)	0	662 (1.3)	−0.03	836 (1.6)	−0.01
White	46 054 (89.2)	48 061 (93.1)	−0.04	48 349 (93.6)	−0.22	46 830 (90.7)	−0.02
Other^b^	743 (1.4)	592 (1.2)	0	598 (1.2)	0.03	667 (1.3)	0
Unknown	176 (0.3)	128 (0.3)	0	122 (0.2)	0.03	174 (0.3)	0
Trauma center proximity							
In county	42 051 (81.4)	42 103 (81.5)	0	40 961 (79.3)	−0.08	41 794 (80.9)	−0.01
Adjacent county	8012 (15.5)	8041 (15.6)		8792 (17.0)		8254 (16.0)	
No trauma center	1575 (3.1)	1494 (2.9)		1885 (3.7)		1590 (3.1)	
AIS score ≥3							
Head and neck	5359 (10.4)	5315 (10.3)	0	5118 (9.9)	0.01	5225 (10.1)	0.01
Face	<10^c^	<10^c^	0.01	<10^c^	0	<10^c^	0
Chest	2634 (5.1)	2576 (5.0)	0	2566 (5.0)	0	2399 (4.7)	0.01
Abdomen and pelvis	343 (0.7)	269 (0.5)	0	303 (0.6)	−0.01	229 (0.4)	0.01
Extremity	24 551 (47.5)	24 507 (47.5)	0	24 714 (47.9)	−0.01	24 515 (47.5)	0
External	89 (0.2)	87 (0.2)	0	89 (0.2)	0.03	88 (0.2)	0.01
Injury subtype							
Cervical spine fracture	733 (1.4)	733 (1.4)	NA	733 (1.4)	NA	733 (1.4)	NA
Hip fracture and TBI	754 (1.5)	754 (1.5)	NA	754 (1.5)	NA	754 (1.5)	NA
Isolated fracture							
Hip	23 827 (46.1)	23 827 (46.1)	NA	23 827 (46.1)	NA	23 827 (46.1)	NA
Rib	2327 (4.5)	2327 (4.5)	NA	2327 (4.5)	NA	2327 (4.5)	NA
Isolated TBI	4575 (8.9)	4575 (8.9)	NA	4575 (8.9)	NA	4575 (8.9)	NA
Other extremity fracture^d^	8725 (16.9)	8725 (16.9)	NA	8725 (16.9)	NA	8725 (16.9)	NA
Other injury^e^	6575 (12.7)	6575 (12.7)	NA	6575 (12.7)	NA	6575 (12.7)	NA
Multiple traumas	4122 (7.9)	4122 (7.9)	NA	4122 (7.9)	NA	4122 (7.9)	NA

Abbreviations: AIS, Abbreviated Injury Scale; CCI, Charlson Comorbidity Index; NA, not applicable; SMD, standardized mean difference; TBI, traumatic brain injury.

^a^ SMD compared with Level I trauma center.

^b^ Other race includes beneficiaries reporting a race category not otherwise listed, including those reporting 2 or more races. Beneficiaries with unknown race are reported separately.

^c^ Data suppressed because sample size was less than 10.

^d^ Other extremity fracture includes isolated fractures of the arm or leg other than hip fractures.

^e^ Other injury includes any National Trauma Data Bank–eligible injury diagnosis not otherwise categorized.

**Figure 2.**
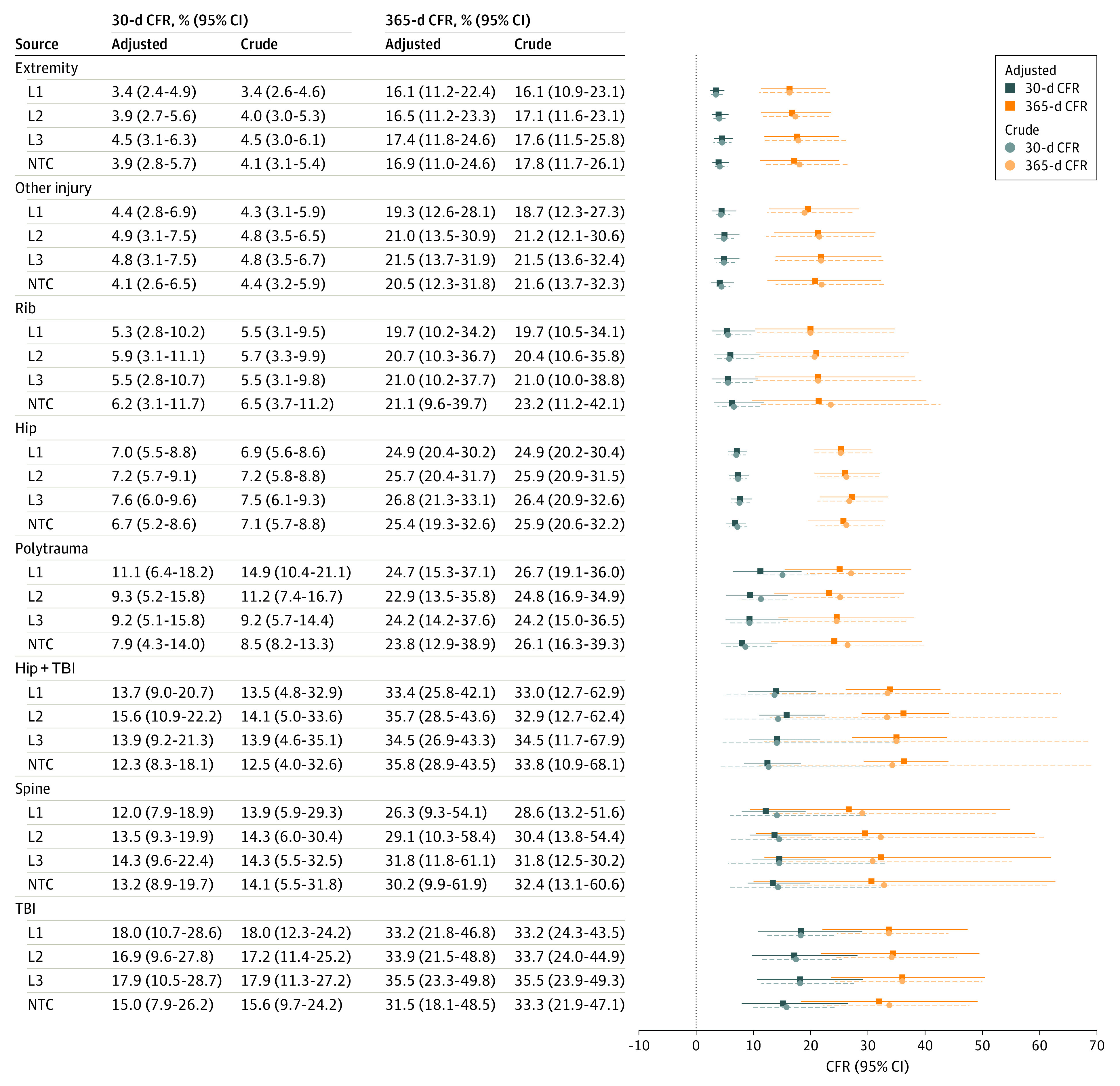
Crude and Adjusted Case Fatality Rates (CFRs) by Injury Type and Trauma Center Level Trauma center levels were level I (L1), level II (L2), level III (L3), and non-trauma center (NTC). Crude CFRs and 95% CIs estimated as the mean marginal probability of death using bivariable logistic regression. Adjusted CFRs estimated as the mean marginal probability of death using hierarchical logistic regression with random effects at the hospital level. Propensity score matched based on age, Abbreviated Injury Scale scores, and trauma center proximity. Multivariable adjustment for sex, race, Charlson Comorbidity Index, and Claims-Based Frailty Index.

Figure 2 presents crude and adjusted CFRs at 30 and 365 days after injury for each injury type at each TC level. Odds ratios (ORs) and 95% CIs comparing mortality at level II TCs, level III TCs, and NTCs with level I TCs are presented in Table 3. Compared with patients treated at NTCs, adjusted relative odds of death within 30 days of injury were higher for patients with hip fractures treated at level III TCs (OR, 1.14; 95% CI, 1.06-1.23), for patients with multiple traumas treated at level I TCs (OR, 1.41; 95% CI, 1.19-1.67), and for patients with isolated TBI treated at level I (OR, 1.29; 95% CI, 1.12-1.47), level II (OR, 1.56; 95% CI, 1.01-1.32), and level III (OR, 1.28; 95% CI, 1.12-1.47) TCs. Relative odds of death within 365 days of injury were higher for patients with isolated TBI treated at level III TCs (OR, 1.27; 95% CI, 1.12-1.43) vs NTCs. When marginal estimates of absolute measures of mortality were compared, no statistically significant differences were found in crude or adjusted CFRs across TC levels for any injury type at either follow-up time. The lowest mortality was observed for patients with isolated extremity fracture, with adjusted CFRs for patients with isolated extremity fracture ranging from 3.4% (95% CI, 2.4%-4.9%) at level I TCs to 4.5% (95% CI, 3.1%-6.3%) at level III TCs 30 days after injury. One year after injury, adjusted CFRs for isolated extremity fracture ranged from 16.1% (95% CI, 11.2%-22.4%) at level I TCs to 17.4% (95% CI, 11.8%-24.6%) at level III TCs. The highest mortality was observed for patients with both hip fracture and TBI, with adjusted CFRs for patients with hip fracture and TBI ranging from 12.3% (95% CI, 8.3%-18.1%) at NTCs to 15.6% (95% CI, 10.9%-22.2%) at level II TCs 30 days after injury. One year after injury, adjusted CFRs for patients with hip fracture and TBI ranged from 33.4% (95% CI, 25.8%-42.1%) at level II TCs to 35.8% (95% CI, 28.9%-43.5%) at NTCs.

**Table 3.** Odds Ratios Comparing 30- and 365-Day Mortality by Trauma Center Level Stratified by Injury Type

Injury type and trauma center level	Odds ratio (95% CI)	
	30 Days	365 Days
**Cervical spine fracture**		
Level I	0.94 (0.65-1.35)	0.86 (0.63-1.18)
Level II	0.97 (0.68-1.38)	0.92 (0.68-1.25)
Level III	1.07 (0.75-1.53)	1.14 (0.84-1.55)
Level IV/NTC	1 [Reference]	1 [Reference]
**Hip fracture and TBI**		
Level I	1.05 (0.74-1.50)	0.82 (0.60-1.13)
Level II	1.32 (0.94-1.86)	0.94 (0.70-1.27)
Level III	1.34 (0.94-1.89)	0.98 (0.72-1.32)
Level IV/NTC	1 [Reference]	1 [Reference]
**Isolated hip fracture**		
Level I	1.03 (0.95-1.11)	0.96 (0.91-1.02)
Level II	1.07 (0.99-1.15)	1.00 (0.95-1.06)
Level III	1.14 (1.06-1.23)	1.07 (1.01-1.13)
Level IV/NTC	1 [Reference]	1 [Reference]
**Isolated rib fracture**		
Level I	0.87 (0.67-1.14)	0.94 (0.79-1.14)
Level II	0.97 (0.75-1.25)	0.99 (0.83-1.18)
Level III	0.87 (0.68-1.13)	0.96 (0.81-1.14)
Level IV/NTC	1 [Reference]	1 [Reference]
**Isolated TBI**		
Level I	1.29 (1.12-1.47)	1.11 (0.98-1.26)
Level II	1.56 (1.01-1.32)	1.12 (1.00-1.27)
Level III	1.28 (1.12-1.47)	1.27 (1.12-1.43)
Level IV/NTC	1 [Reference]	1 [Reference]
**Other extremity fracture^a^**		
Level I	0.86 (0.73-1.01)	0.93 (0.84-1.03)
Level II	0.98 (0.83-1.14)	0.98 (0.89-1.08)
Level III	1.09 (0.93-1.27)	1.02 (0.93-1.12)
Level IV/NTC	1 [Reference]	1 [Reference]
**Other injury^b^**		
Level I	1.04 (0.87-1.24)	0.91 (0.81-1.01)
Level II	1.17 (0.98-1.39)	1.01 (0.91-1.12)
Level III	1.17 (0.98-1.38)	1.05 (0.95-1.16)
Level IV/NTC	1 [Reference]	1 [Reference]
**Multiple traumas**		
Level I	1.41 (1.19-1.67)	1.09 (0.95-1.25)
Level II	1.18 (0.99-1.40)	0.96 (0.84-1.09)
Level III	1.10 (0.93-1.31)	1.01 (0.89-1.15)
Level IV/NTC	1 [Reference]	1 [Reference]

Abbreviations: NTC, non-trauma center; TBI, traumatic brain injury.

^a^ Other extremity fracture includes isolated fractures of the arm or leg other than hip fractures.

^b^ Other injury includes any National Trauma Data Bank eligible injury diagnosis not otherwise categorized.

## Discussion

In this large, nationally representative cohort study of older adults with traumatic injury, TC level was not associated with postdischarge survival after adjustment for injury characteristics, preinjury health status, and demographic characteristics. This finding is consistent with our hypothesis that older adults with injuries from low-energy blunt mechanisms (ie, isolated hip fracture) would not benefit from TC care. We also found no clinically significant association between TC level and long-term survival for older adults with injuries that do typically benefit from treatment at a level I or II TC in younger populations,^5^ including multiple traumas, TBI, and spinal cord injuries. Previous studies of trauma care for injured older adults have been limited by small samples size,^5^ lack of geographic representation,^6,7,8,24,25^ and inadequate adjustment for preinjury health status.^8,24,25^ Our work addresses a critical gap in the understanding of geriatric trauma care effectiveness by examining short- and long-term trauma mortality after injuries most common among older adults, regardless of hospital- and state-level criteria for trauma registry inclusion.

One-year CFRs of 22% to 24% after multiple traumas, 26% to 32% after cervical spine fracture, and 25% to 27% after isolated hip fractures demonstrated in our analyses are consistent with previous studies^5,26^ of long-term mortality from these injuries. Our findings also demonstrate long-term mortality outcomes after other injuries common in older adults, including nonhip extremity fractures (1-year CFR, 16%-17%) and isolated rib fracture (1-year CFR, 19%-21%), which have not been previously published. The variability in long-term mortality across injury types observed in this study illustrates the need to disaggregate studies of older adult trauma outcomes by injury type when examining trauma system interventions that address geriatric trauma care. Although the lack of association between TC care and mortality was consistent across injury types, reasons for this likely differ by injury type. For isolated injuries, such as hip fracture, treatment at a TC may increase the risk of treatment delays,^27^ which in turn increases the risk of poor outcomes.^28^ For multiple traumas, the lack of association may be because severe, complex injuries are a seminal health event, resulting in high risk of death regardless of immediate lifesaving interventions.

The consistency in mortality outcomes across TC levels highlights the need to consider outcomes other than mortality when developing guidelines for triage of older patients with trauma. Although TC care does not appear to confer a survival advantage for injured older adults, it is possible that TC care may result in better end-of-life care among older adults who die as a result of their injury and/or improved quality of life and functional status among older adults who survive long term after injury. Alternatively, triage of older patients with trauma to TCs farther from home may increase out-of-pocket costs for patients and families and may have deleterious health effects if triage decisions create discontinuity in management of existing chronic illness. Hospital-level effects of triage decisions should also be examined because systematic triage of submajor trauma away from smaller community hospitals may negatively affect financial viability of these hospitals.

Although national guidelines direct EMS personnel to transport more severely injured patients to TCs,^2^ our adjusted findings suggest that geographic proximity to a TC has a significant influence on level of TC care for older adults. This finding is consistent with substantial evidence suggesting that triage guidelines do not heavily influence hospital destination for older adults, including evidence that prehospital triage and destination decisions for injured older adults are most often based on family and/or patient choice or hospital proximity,^29^ prehospital physiologic stability is a poor indicator of triage decisions and hospital destination,^30^ and half of all severely injured older adults are treated at NTCs.^31,32^ Furthermore, EMS personnel report that they frequently make triage decisions before assessing physiologic stability, most often relying on anatomical injury characteristics and observation of injury mechanism when making triage decisions.^33^ Prior studies^5,7,8^ have addressed this potential bias by controlling for prehospital physiologic measures. Such measures are not available in Medicare data. Documented patterns of EMS triage^29,30,33^ suggest that the threat of confounding by indication in the older adult population can be addressed by controlling for anatomical injury characteristics. This decision process is substantiated by our findings that older adults with anatomical injury characteristics specifically directed to TC care in national triage guidelines^2^ (ie, multiple traumas, TBI, or severe chest injury) are more likely to be treated at level I or II TCs, whereas older adults with injuries for which physiologic measures are better indicators of potential instability (ie, severe isolated extremity injuries) are more likely to be treated at NTCs.

### Limitations

This study has several limitations. Non-trauma centers may underdiagnose injuries compared with TCs because of injury survey practices. Such bias in diagnosis is likely to occur in cases of early trauma death, when TCs must fully assess injury severity for trauma registry reporting. Consistent with previous studies^5,6,8^ of TC effectiveness, our study excluded early trauma deaths, minimizing concerns about underdiagnosis. Residual bias may be present after propensity score matching because of unmeasured prehospital physiologic stability. Previous studies^5,6,7,8^ of TC effectiveness for older adults relied on trauma registries, the only readily available data sets with prehospital measures for patients with trauma. Registries represent only 40% of seriously injured older adults and 20% of older adult injury deaths,^34^ severely limiting generalizability and offsetting methodologic gains from adjustment for physiologic stability. In contrast, we used an administrative data resource that represents 50% of US older adults regardless of injury type or treatment location and is generalizable to the 98% of older adults insured through Medicare.^35^ We minimized threats from confounding by using nationally representative, population-based data, stratifying the cohort by anatomical injury type, and propensity score matching to balance observed variables known to influence triage decisions within each injury type. Our analysis assumed that multiple same-day or sequential-day encounters for injury represented transfers and patients moved from lowest level of care (NTC) to highest level (TC). Under this algorithm, patients who experienced delayed transfers, such as when an NTC holds a patient for more than 24 hours before transfer to a TC, are attributed to the NTC. This approach may overrepresent mortality for patients who receive care at NTCs and underrepresent mortality for TC patients, thus overestimating benefits of TC care and underestimating benefits of NTC care. Because of costs and extended timelines for acquisition of Medicare claims data, our analyses used data for health care encounters in 2014 to 2015. No significant changes to geriatric trauma care guidelines of policies have occurred in the intervening years.

## Conclusions

Older adults account for most injury-related hospital admissions and deaths in the US, yet the results of this cohort study suggest that they do not benefit from existing TC care as younger adults do. Current guidelines for geriatric trauma care are built on the presumption that benefits of TC care are consistent across the life span. As the US trauma care system moves to develop and implement protocols for geriatric trauma care,^36^ we must understand long-term outcomes of TC care in the older adult population and identify the most effective practices for managing injuries from all mechanisms and including all anatomical patterns.

## Notes

Supplement. **eTable 1.** National Trauma Data Standard ICD Codes **eTable 2.** Descriptive Statistics by Trauma Level, Cervical Spine Fracture **eTable 3.** Descriptive Statistics by Trauma Level, Hip Fracture, and Traumatic Brain Injury **eTable 4.** Descriptive Statistics by Trauma Level, Isolated Hip Fracture **eTable 5.** Descriptive Statistics by Trauma Level, Isolated Rib Fracture **eTable 6.** Descriptive Statistics by Trauma Level, Isolated Traumatic Brain Injury **eTable 7.** Descriptive Statistics by Trauma Level, Other Isolated Extremity Fracture **eTable 8.** Descriptive Statistics by Trauma Level, Other Injuries **eTable 9.** Descriptive Statistics by Trauma Level, Polytrauma **eFigure.** Standardized Mean Differences for Matching Variables, Before and After Propensity Score Matching
