# Supplementary material for: Association of Trauma Center Designation With Postdischarge Survival Among Older Adults With Injuries
Source: JAMA Netw Open. 2022 Mar 16;5(3):e222448. doi: 10.1001/jamanetworkopen.2022.2448 (PMC8928003; doi:10.1001/jamanetworkopen.2022.2448)
Supplement: Supplement. — eTable 1. National Trauma Data Standard ICD Codes eTable 2. Descriptive Statistics by Trauma Level, Cervical Spine Fracture eTable 3. Descriptive Statistics by Trauma Level, Hip Fracture, and Traumatic Brain Injury eTable 4. Descriptive Statistics by Trauma Level, Isolated Hip Fracture eTable 5. Descriptive Statistics by Trauma Level, Isolated Rib Fracture eTable 6. Descriptive Statistics by Trauma Level, Isolated Traumatic Brain Injury eTable 7. Descriptive Statistics by Trauma Level, Other Isolated Extremity Fracture eTable 8. Descriptive Statistics by Trauma Level, Other Injuries eTable 9. Descriptive Statistics by Trauma Level, Polytrauma eFigure. Standardized Mean Differences for Matching Variables, Before and After Propensity Score Matching [file jamanetwopen-e222448-s001.pdf]

Supplementary Online Content

Jarman MP, Jin G, Weissman JS, et al. Association of trauma center designation with postdischarge survival among older adults with injuries. *JAMA Netw Open*. 2022;5(3):e222448. doi:10.1001/jamanetworkopen.2022.2448

eTable 1. National Trauma Data Standard ICD Codes
eTable 2. Descriptive Statistics by Trauma Level, Cervical Spine Fracture
eTable 3. Descriptive Statistics by Trauma Level, Hip Fracture, and Traumatic Brain Injury
eTable 4. Descriptive Statistics by Trauma Level, Isolated Hip Fracture
eTable 5. Descriptive Statistics by Trauma Level, Isolated Rib Fracture
eTable 6. Descriptive Statistics by Trauma Level, Isolated Traumatic Brain Injury
eTable 7. Descriptive Statistics by Trauma Level, Other Isolated Extremity Fracture
eTable 8. Descriptive Statistics by Trauma Level, Other Injuries
eTable 9. Descriptive Statistics by Trauma Level, Polytrauma
eFigure. Standardized Mean Differences for Matching Variables, Before and After Propensity Score Matching

This supplementary material has been provided by the authors to give readers additional information about their work.

eTable 1: National Trauma Data Standard ICD codes

Code Standard	Included Range	Excluded
**ICD9**	800-959.9<br>905-909<br>910-924<br>930-939	Superficial injury<br>Foreign Body in Orifice<br>Late Effects of Injury
**ICD10**	S00-S99<br>T07<br>T14<br>T20-T28<br>T30-T32<br>T79	

© 2022 Jarman MP et al. *JAMA Network Open.*

eTable 2: Descriptive statistics by trauma level, cervical spine fracture

	Full Population						
**Cervical Spine Fracture**	**Level I**	**Level II**		**Level III**		**Non-Trauma**	
**Patient Characteristics**	**No. (%)**	**No. (%)**	**SMD[a]**	**No. (%)**	**SMD[a]**	**No. (%)**	**SMD[a]**
**Total (N)**	1,664	2,097		733		1,991	
**Age**							
**65-74**	368 (22.1)	380 (18.1)	-0.18	130 (17.7)	-0.16	277 (13.9)	-0.30
**75-84**	578 (34.7)	697 (33.2)		245 (33.4)		623 (31.3)	
**≥85**	718 (43.1)	1,020 (48.6)		358 (48.8)		1,091 (54.8)	
**CCI**							
**0**	543 (32.6)	653 (31.1)	0.01	200 (27.3)	-0.13	660 (33.1)	0.00
**1**	399 (24.0)	537 (25.6)		183 (25.0)		466 (23.4)	
**2**	272 (16.3)	351 (16.7)		115 (15.7)		320 (16.1)	
**≥3**	450 (27.0)	556 (26.5)		235 (32.1)		545 (27.4)	
**Frail**	874 (52.5)	1,142 (54.5)	-0.05	387 (52.8)	-0.01	1,153 (57.9)	-0.11
**Female**	899 (54.0)	1,183 (56.4)	-0.07	401 (54.7)	-0.02	1,210 (60.8)	-0.14
**Race**							
**White**	1,447 (87.5)	1,959 (93.7)	-0.31	682 (96.2)	-0.29	1,808 (91.0)	-0.12
**Black**	136 (8.2)	69 (3.3)	0.30	27 (3.8)	0.27	85 (4.3)	0.16
**Asian**	26 (1.6)	24 (1.1)	0.08	0 (0.0)	0.07	28 (1.4)	-0.05
**Hispanic**	25 (1.5)	19 (0.9)	0.03	0 (0.0)	0.01	43 (2.2)	0.00
**Other**	20 (1.2)	20 (1.0)	0.05	0 (0.0)	0.06	23 (1.2)	0.01
**Unknown**	0 (0.0)	0 (0.0)	N/A	0 (0.0)	N/A	0 (0.0)	N/A
**Trauma Center Proximity**							
**In county**	1,322 (79.4)	1,561 (74.4)	0.07	524 (71.5)	0.11	1,260 (63.3)	0.06
**Adjacent county**	277 (16.6)	417 (19.9)		158 (21.6)		555 (27.9)	
**No trauma center**	65 (3.9)	119 (5.7)		51 (7.0)		176 (8.8)	
**AIS Head/Neck ≥3**	546 (32.8)	499 (23.8)	-0.18	145 (19.8)	-0.28	342 (17.2)	-0.36
**AIS Face ≥3**	0 (0.0)	0 (0.0)	N/A	0 (0.0)	N/A	0 (0.0)	N/A
**AIS Chest ≥3**	0 (0.0)	0 (0.0)	N/A	0 (0.0)	N/A	0 (0.0)	N/A
**AIS Abdomen/Pelvic ≥3**	0 (0.0)	0 (0.0)	N/A	0 (0.0)	N/A	0 (0.0)	N/A
**AIS Extremity ≥3**	0 (0.0)	0 (0.0)	N/A	0 (0.0)	N/A	0 (0.0)	N/A
**AIS External ≥3**	0 (0.0)	0 (0.0)	N/A	0 (0.0)	N/A	0 (0.0)	N/A

© 2022 Jarman MP et al. *JAMA Network Open.*

	After Propensity Score Matching						
	Level I	Level II		Level III		Non-Trauma	
**Patient Characteristics**	**No. (%)**	**No. (%)**	**SMD[a]**	**No. (%)**	**SMD[a]**	**No. (%)**	**SMD[a]**
**Total (N)**	733	733		733		733	
**Age**							
**65-74**	131 (17.9)	131 (17.9)	0.00	130 (17.7)	0.00	131 (17.9)	0.00
**75-84**	247 (33.7)	247 (33.7)		245 (33.4)		247 (33.7)	
**≥85**	355 (48.4)	355 (48.4)		358 (48.8)		355 (48.4)	
**CCI**							
**0**	244 (33.3)	248 (33.8)	-0.02	200 (27.3)	-0.18	240 (32.7)	-0.06
**1**	185 (25.2)	177 (24.1)		183 (25.0)		182 (24.8)	
**2**	119 (16.2)	116 (15.8)		115 (15.7)		110 (15.0)	
**≥3**	185 (25.2)	192 (26.2)		235 (32.1)		201 (27.4)	
**Frail**	376 (51.3)	408 (55.7)	-0.12	387 (52.8)	-0.04	417 (56.9)	-0.11
**Female**	422 (57.6)	431 (58.8)	-0.04	401 (54.7)	0.08	451 (61.5)	-0.08
**Race**							
**White**	644 (90.3)	678 (95.1)	-0.22	682 (96.2)	-0.25	663 (93.0)	-0.08
**Black**	53 (7.4)	24 (3.4)	0.25	27 (3.8)	0.22	34 (4.8)	0.11
**Asian**	16 (2.2)	11 (1.5)	-0.06	0 (0.0)	-0.02	0 (0.0)	-0.11
**Hispanic**	0 (0.0)	0 (0.0)	0.04	0 (0.0)	0.04	16 (2.2)	0.04
**Other**	0 (0.0)	0 (0.0)	N/A	0 (0.0)	N/A	0 (0.0)	N/A
**Unknown**	0 (0.0)	0 (0.0)	N/A	0 (0.0)	N/A	0 (0.0)	N/A
**Trauma Center Proximity**							
**In county**	560 (76.4)	567 (77.4)	0.07	524 (71.5)	0.12	555 (75.7)	-0.01
**Adjacent county**	142 (19.4)	138 (18.8)		158 (21.6)		147 (20.1)	
**No trauma center**	31 (4.2)	28 (3.8)		51 (7.0)		31 (4.2)	
**AIS Head/Neck ≥3**	151 (20.6)	146 (19.9)	0.03	145 (19.8)	0.10	133 (18.1)	0.03
**AIS Face ≥3**	0 (0.0)	0 (0.0)	N/A	0 (0.0)	N/A	0 (0.0)	N/A
**AIS Chest ≥3**	0 (0.0)	0 (0.0)	N/A	0 (0.0)	N/A	0 (0.0)	N/A
**AIS Abdomen/Pelvic ≥3**	0 (0.0)	0 (0.0)	N/A	0 (0.0)	N/A	0 (0.0)	N/A
**AIS Extremity ≥3**	0 (0.0)	0 (0.0)	N/A	0 (0.0)	N/A	0 (0.0)	N/A
**AIS External ≥3**	0 (0.0)	0 (0.0)	N/A	0 (0.0)	N/A	0 (0.0)	N/A

[a]SMD: Standardized mean difference, compared to Level I Trauma Center
[b]Other race includes beneficiaries reporting a race category not otherwise listed, include those reporting two or more races. Beneficiaries with unknown race are reported separately.

© 2022 Jarman MP et al. *JAMA Network Open.*

eTable 3: Descriptive statistics by trauma level, hip fracture and traumatic brain injury

	Full Population						
**Hip Fracture and TBI**	**Level I**	**Level II**		**Level III**		**Non-Trauma**	
**Patient Characteristics**	**No. (%)**	**No. (%)**	**SMD[a]**	**No. (%)**	**SMD[a]**	**No. (%)**	**SMD[a]**
**Total (N)**	830	1,359		754		2,122	
**Age**							
**65-74**	120 (14.5)	160 (11.8)	-0.15	76 (10.1)	-0.16	202 (9.5)	-0.16
**75-84**	254 (30.6)	432 (31.8)		236 (31.3)		642 (30.3)	
**≥85**	456 (54.9)	767 (56.4)		442 (58.6)		1,278 (60.2)	
**CCI**							
**0**	243 (29.3)	410 (30.2)	0.06	235 (31.2)	0.05	649 (30.6)	0.06
**1**	197 (23.7)	317 (23.3)		173 (22.9)		513 (24.2)	
**2**	158 (19.0)	234 (17.2)		132 (17.5)		385 (18.1)	
**≥3**	232 (28.0)	398 (29.3)		214 (28.4)		575 (27.1)	
**Frail**	469 (56.5)	825 (60.7)	-0.12	463 (61.4)	-0.14	1,343 (63.3)	-0.14
**Female**	563 (67.8)	955 (70.3)	-0.07	537 (71.2)	-0.10	1,580 (74.5)	-0.15
**Race**							
**White**	753 (91.3)	1,292 (95.1)	-0.24	708 (94.1)	-0.17	1,981 (93.4)	-0.10
**Black**	46 (5.6)	23 (1.7)	0.29	16 (2.1)	0.25	51 (2.4)	0.16
**Asian**	0 (0.0)	14 (1.0)	0.03	0 (0.0)	0.06	16 (0.8)	-0.01
**Hispanic**	13 (1.6)	18 (1.3)	0.09	0 (0.0)	0.00	36 (1.7)	-0.01
**Other**	13 (1.6)	12 (0.9)	-0.07	12 (1.6)	-0.07	37 (1.7)	-0.02
**Unknown**	0 (0.0)	0 (0.0)	N/A	0 (0.0)	N/A	0 (0.0)	N/A
**Trauma Center Proximity**							
**In county**	675 (81.3)	1,027 (75.6)	-0.21	552 (73.2)	-0.10	1,256 (59.2)	-0.49
**Adjacent county**	131 (15.8)	268 (19.7)		156 (20.7)		677 (31.9)	
**No trauma center**	24 (2.9)	64 (4.7)		46 (6.1)		189 (8.9)	
**AIS Head/Neck ≥3**	370 (44.6)	483 (35.5)	0.25	169 (22.4)	-0.30	522 (24.6)	0.38
**AIS Face ≥3**	0 (0.0)	0 (0.0)	N/A	0 (0.0)	N/A	0 (0.0)	N/A
**AIS Chest ≥3**	0 (0.0)	0 (0.0)	N/A	0 (0.0)	N/A	0 (0.0)	N/A
**AIS Abdomen/Pelvic ≥3**	0 (0.0)	0 (0.0)	N/A	0 (0.0)	N/A	0 (0.0)	N/A
**AIS Extremity ≥3**	553 (66.6)	912 (67.1)	0.03	567 (75.2)	-0.19	1,562 (73.6)	-0.13
**AIS External ≥3**	0 (0.0)	0 (0.0)	N/A	0 (0.0)	N/A	0 (0.0)	N/A

© 2022 Jarman MP et al. *JAMA Network Open.*

	After Propensity Score Matching						
	Level I	Level II		Level III		Non-Trauma	
Patient Characteristics	No. (%)	No. (%)	SMD[a]	No. (%)	SMD[a]	No. (%)	SMD[a]
Total (N)	754	754		754		754	
Age							
65-74	75 (9.9)	76 (10.1)	0.00	76 (10.1)	0.00	78 (10.3)	0.00
75-84	234 (31.0)	234 (31.0)		236 (31.3)		229 (30.4)	
≥85	445 (59.0)	444 (58.9)		442 (58.6)		447 (59.3)	
CCI							
0	222 (29.4)	224 (29.7)	0.02	235 (31.2)	0.03	223 (29.6)	0.08
1	178 (23.6)	177 (23.5)		173 (22.9)		199 (26.4)	
2	146 (19.4)	137 (18.2)		132 (17.5)		140 (18.6)	
≥3	208 (27.6)	216 (28.6)		214 (28.4)		192 (25.5)	
Frail	433 (57.4)	448 (59.4)	-0.06	463 (61.4)	-0.11	475 (63.0)	-0.11
Female	522 (69.2)	529 (70.2)	-0.03	537 (71.2)	-0.06	541 (71.8)	-0.06
Race							
White	687 (91.6)	715 (96.4)	-0.21	708 (96.2)	-0.15	701 (93.8)	-0.07
Black	37 (4.9)	16 (2.2)	0.21	16 (2.2)	0.21	20 (2.7)	0.12
Asian	0 (0.0)	0 (0.0)	0.03	0 (0.0)	0.08	0 (0.0)	0.02
Hispanic	13 (1.7)	11 (1.5)	0.10	0 (0.0)	0.01	11 (1.5)	-0.02
Other	13 (1.7)	0 (0.0)	-0.02	12 (1.6)	-0.08	15 (2.0)	-0.05
Unknown	0 (0.0)	0 (0.0)	N/A	0 (0.0)	N/A	0 (0.0)	N/A
Trauma Center Proximity							
In county	626 (83.0)	630 (83.6)	0.06	552 (73.2)	-0.35	610 (80.9)	-0.03
Adjacent county	106 (14.1)	113 (15.0)		156 (20.7)		126 (16.7)	
No trauma center	22 (2.9)	11 (1.5)		46 (6.1)		18 (2.4)	
AIS Head/Neck ≥3	308 (40.8)	279 (37.0)	0.07	169 (22.4)	0.53	260 (34.5)	0.09
AIS Face ≥3	0 (0.0)	0 (0.0)	N/A	0 (0.0)	N/A	0 (0.0)	N/A
AIS Chest ≥3	0 (0.0)	0 (0.0)	N/A	0 (0.0)	N/A	0 (0.0)	N/A
AIS Abdomen/Pelvic ≥3	0 (0.0)	0 (0.0)	N/A	0 (0.0)	N/A	0 (0.0)	N/A
AIS Extremity ≥3	516 (68.4)	522 (69.2)	-0.01	567 (75.2)	-0.12	521 (69.1)	-0.01
AIS External ≥3	0 (0.0)	0 (0.0)	N/A	0 (0.0)	N/A	0 (0.0)	N/A

[a]SMD: Standardized mean difference, compared to Level I Trauma Center
[b]Other race includes beneficiaries reporting a race category not otherwise listed, include those reporting two or more races. Beneficiaries with unknown race are reported separately.

© 2022 Jarman MP et al. *JAMA Network Open.*

eTable 4: Descriptive statistics by trauma level, isolated hip fracture

	Full Population						
**Isolated Hip Fracture**	**Level I**	**Level II**		**Level III**		**Non-Trauma**	
**Patient Characteristics**	**No. (%)**	**No. (%)**	**SMD[a]**	**No. (%)**	**SMD[a]**	**No. (%)**	**SMD[a]**
**Total (N)**	23,827	40,363		34,244		115,471	
**Age**							
**65-74**	3,570 (15.0)	5,651 (14.0)	-0.03	5,045 (14.7)	0.04	15,565 (13.5)	-0.03
**75-84**	7,770 (32.6)	13,480 (33.4)		11,990 (35.0)		38,326 (33.2)	
**≥85**	12,487 (52.4)	21,232 (52.6)		17,209 (50.3)		61,580 (53.3)	
**CCI**							
**0**	7,761 (32.6)	13,293 (32.9)	0.04	10,997 (32.1)	0.04	38,531 (33.4)	0.06
**1**	5,400 (22.7)	9,442 (23.4)		8,240 (24.1)		27,977 (24.2)	
**2**	4,012 (16.8)	6,749 (16.7)		5,706 (16.7)		19,284 (16.7)	
**≥3**	6,654 (27.9)	10,879 (26.9)		9,301 (27.2)		29,679 (25.7)	
**Frail**	12,489 (52.4)	21,265 (52.7)	-0.01	17,899 (52.3)	0.00	60,647 (52.5)	0.00
**Female**	17,247 (72.4)	29,597 (73.3)	-0.03	25,094 (73.3)	-0.03	85,228 (73.8)	-0.03
**Race**							
**White**	21,604 (90.7)	38,071 (94.3)	-0.20	32,326 (94.4)	-0.20	107,535 (93.1)	-0.09
**Black**	1,290 (5.4)	1,100 (2.7)	0.19	893 (2.6)	0.20	3,788 (3.3)	0.10
**Asian**	279 (1.2)	392 (0.9)	0.08	394 (1.2)	0.14	1,123 (0.9)	0.02
**Hispanic**	366 (1.5)	359 (0.9)	0.00	186 (0.5)	-0.02	1,450 (1.3)	-0.02
**Other**	225 (0.9)	382 (0.9)	0.03	382 (1.1)	0.00	1,356 (1.2)	0.02
**Unknown**	63 (0.3)	59 (0.2)	0.04	63 (0.2)	0.02	219 (0.2)	0.02
**Trauma Center Proximity**							
**In county**	20,001 (83.9)	30,187 (74.8)	-0.34	25,198 (73.6)	-0.38	64,706 (56.0)	-0.63
**Adjacent county**	3,287 (13.8)	8,050 (19.9)		7,078 (20.7)		38,534 (33.4)	
**No trauma center**	539 (2.3)	2,126 (5.3)		1,968 (5.8)		12,231 (10.6)	
**AIS Head/Neck ≥3**	0 (0.0)	0 (0.0)	N/A	0 (0.0)	N/A	0 (0.0)	N/A
**AIS Face ≥3**	0 (0.0)	0 (0.0)	N/A	0 (0.0)	N/A	0 (0.0)	N/A
**AIS Chest ≥3**	0 (0.0)	0 (0.0)	N/A	0 (0.0)	N/A	0 (0.0)	N/A
**AIS Abdomen/Pelvic ≥3**	0 (0.0)	0 (0.0)	N/A	0 (0.0)	N/A	0 (0.0)	N/A
**AIS Extremity ≥3**	20,665 (86.7)	35,052 (86.8)	0.00	30,369 (88.7)	-0.07	100,469 (87.0)	-0.01
**AIS External ≥3**	0 (0.0)	0 (0.0)	N/A	0 (0.0)	N/A	0 (0.0)	N/A

© 2022 Jarman MP et al. *JAMA Network Open.*

	After Propensity Score Matching						
	Level I	Level II		Level III		Non-Trauma	
Patient Characteristics	No. (%)	No. (%)	SMD[a]	No. (%)	SMD[a]	No. (%)	SMD[a]
Total (N)	23,827	23,827		23,827		23,827	
Age							
65-74	3,570 (14.9)	3,570 (14.9)	0.00	3,568 (14.9)	0.00	3,569 (14.9)	0.00
75-84	7,770 (32.6)	7,770 (32.6)		7,774 (32.6)		7,771 (32.6)	
≥85	12,487 (52.4)	12,487 (52.4)		12,485 (52.4)		12,487 (52.4)	
CCI							
0	7,761 (32.6)	7,847 (32.9)	0.04	7,669 (32.2)	0.05	8,115 (34.1)	0.07
1	5,400 (22.7)	5,573 (23.4)		5,720 (24.0)		5,743 (24.1)	
2	4,012 (16.8)	4,007 (16.8)		4,007 (16.8)		3,927 (16.5)	
≥3	6,654 (27.9)	6,400 (26.9)		6,431 (26.9)		6,042 (25.4)	
Frail	12,489 (52.4)	12,489 (52.4)	0.00	12,482 (52.4)	0.00	12,524 (52.6)	0.00
Female	17,247 (72.4)	17,439 (73.2)	-0.03	17,566 (73.7)	-0.04	17,457 (73.3)	-0.02
Race							
White	21,604 (90.7)	22,413 (94.1)	-0.18	22,506 (94.5)	-0.20	22,003 (92.3)	-0.06
Black	1,290 (5.4)	662 (2.8)	0.19	634 (2.7)	0.20	802 (3.4)	0.10
Asian	279 (1.2)	229 (0.9)	0.07	240 (1.0)	0.15	255 (1.1)	0.00
Hispanic	366 (1.5)	242 (1.0)	-0.01	108 (0.5)	-0.04	357 (1.5)	-0.05
Other	225 (0.9)	242 (1.0)	0.03	292 (1.2)	0.02	347 (1.5)	0.01
Unknown	63 (0.3)	39 (0.2)	0.03	47 (0.2)	0.02	63 (0.3)	0.00
Trauma Center Proximity							
In county	20,001 (83.9)	19,979 (83.9)	0.00	19,936 (83.7)	-0.01	19,983 (83.9)	0.00
Adjacent county	3,287 (13.8)	3,304 (13.9)		3,351 (14.1)		3,304 (13.9)	
No trauma center	539 (2.3)	544 (2.3)		540 (2.3)		540 (2.3)	
AIS Head/Neck ≥3	0 (0.0)	0 (0.0)	N/A	0 (0.0)	N/A	0 (0.0)	N/A
AIS Face ≥3	0 (0.0)	0 (0.0)	N/A	0 (0.0)	N/A	0 (0.0)	N/A
AIS Chest ≥3	0 (0.0)	0 (0.0)	N/A	0 (0.0)	N/A	0 (0.0)	N/A
AIS Abdomen/Pelvic ≥3	0 (0.0)	0 (0.0)	N/A	0 (0.0)	N/A	0 (0.0)	N/A
AIS Extremity ≥3	20,665 (86.7)	20,686 (86.8)	0.00	20,766 (87.2)	-0.01	20,670 (86.8)	0.00
AIS External ≥3	0 (0.0)	0 (0.0)	N/A	0 (0.0)	N/A	0 (0.0)	N/A

[a]SMD: Standardized mean difference, compared to Level I Trauma Center
[b]Other race includes beneficiaries reporting a race category not otherwise listed, include those reporting two or more races. Beneficiaries with unknown race are reported separately.

© 2022 Jarman MP et al. *JAMA Network Open.*

eTable 5: Descriptive statistics by trauma level, isolated rib fracture

	Full Population						
**Isolated Rib Fracture**	**Level I**	**Level II**		**Level III**		**Non-Trauma**	
**Patient Characteristics**	**No. (%)**	**No. (%)**	**SMD[a]**	**No. (%)**	**SMD[a]**	**No. (%)**	**SMD[a]**
**Total (N)**	3,058	3,838		2,327		6,621	
**Age**							
**65-74**	738 (24.1)	873 (22.7)	-0.06	536 (23.0)	-0.02	1,256 (19.0)	-0.18
**75-84**	1,046 (34.2)	1,294 (33.7)		792 (34.0)		2,085 (31.5)	
**≥85**	1,274 (41.7)	1,671 (43.5)		999 (42.9)		3,280 (49.5)	
**CCI**							
**0**	1,006 (32.9)	1,186 (30.9)	-0.04	657 (28.2)	-0.06	1,933 (29.2)	-0.05
**1**	762 (24.9)	993 (25.9)		615 (26.4)		1,640 (24.8)	
**2**	491 (16.1)	628 (16.4)		405 (17.4)		1,158 (17.5)	
**≥3**	799 (26.1)	1,031 (26.9)		650 (27.9)		1,890 (28.5)	
**Frail**	1,480 (48.4	1,839 (47.9)	0.01	1,155 (49.6)	-0.03	3,537 (53.4)	-0.10
**Female**	1,634 (53.4)	2,079 (54.2)	-0.02	1,234 (53.0)	0.01	3,907 (59.0)	-0.11
**Race**							
**White**	2,747 (90.1)	3,599 (93.8)	-0.20	2,220 (95.6)	-0.30	6,138 (92.7)	-0.10
**Black**	163 (5.3)	106 (2.8)	0.18	37 (1.6)	0.29	200 (3.0)	0.12
**Asian**	48 (1.6)	39 (1.0)	0.07	25 (1.1)	0.16	76 (1.1)	0.03
**Hispanic**	55 (1.8)	47 (1.2)	0.03	13 (0.6)	0.00	97 (1.5)	-0.01
**Other**	35 (1.1)	35 (0.9)	0.07	26 (1.1)	0.06	86 (1.3)	0.04
**Unknown**	0 (0.0)	12 (0.3)	0.00	0 (0.0)	0.02	24 (0.4)	-0.01
**Trauma Center Proximity**							
**In county**	2,437 (79.7)	2,949 (76.8)	-0.10	1,741 (74.8)	-0.17	3,776 (57.0)	-0.49
**Adjacent county**	501 (16.4)	708 (18.4)		461 (19.8)		2,147 (32.4)	
**No trauma center**	120 (3.9)	181 (4.7)		125 (5.4)		698 (10.5)	
**AIS Head/Neck ≥3**	0 (0.0)	0 (0.0)	N/A	0 (0.0)	N/A	0 (0.0)	N/A
**AIS Face ≥3**	0 (0.0)	0 (0.0)	N/A	0 (0.0)	N/A	0 (0.0)	N/A
**AIS Chest ≥3**	1,863 (60.9)	2,171 (56.6)	0.15	1,369 (58.8)	0.12	3,363 (50.8)	0.26
**AIS Abdomen/Pelvic ≥3**	0 (0.0)	0 (0.0)	N/A	0 (0.0)	N/A	0 (0.0)	N/A
**AIS Extremity ≥3**	0 (0.0)	0 (0.0)	N/A	0 (0.0)	N/A	0 (0.0)	N/A
**AIS External ≥3**	0 (0.0)	0 (0.0)	N/A	0 (0.0)	N/A	0 (0.0)	N/A

© 2022 Jarman MP et al. *JAMA Network Open.*

	After Propensity Score Matching						
	Level I	Level II		Level III		Non-Trauma	
**Patient Characteristics**	**No. (%)**	**No. (%)**	**SMD[a]**	**No. (%)**	**SMD[a]**	**No. (%)**	**SMD[a]**
**Total (N)**	2,327	2,327		2,327		2,327	
**Age**							
**65-74**	539 (23.2)	539 (23.2)	0.00	536 (23.0)	0.00	540 (23.2)	0.00
**75-84**	789 (33.9)	788 (33.9)		792 (34.0)		787 (33.8)	
**≥85**	999 (42.9)	1,000 (43.0)		999 (42.9)		1,000 (43.0)	
**CCI**							
**0**	774 (33.3)	728 (31.3)	-0.03	657 (28.2)	-0.07	719 (30.9)	-0.01
**1**	577 (24.8)	606 (26.0)		615 (26.4)		586 (25.2)	
**2**	376 (16.2)	378 (16.2)		405 (17.4)		409 (17.6)	
**≥3**	600 (25.8)	615 (26.4)		650 (27.9)		613 (26.3)	
**Frail**	1,134 (48.7)	1,076 (46.2)	0.07	1,155 (49.6)	-0.03	1,182 (50.8)	-0.04
**Female**	1,220 (52.4)	1,234 (53.0)	0.04	1,296 (55.7)	0.02	1,250 (53.7)	-0.04
**Race**							
**White**	2,092 (90.2)	2,181 (94.0)	-0.20	2,220 (95.6)	-0.30	2,138 (91.9)	-0.07
**Black**	129 (5.6)	60 (2.6)	0.21	37 (1.6)	0.30	73 (3.1)	0.12
**Asian**	39 (1.7)	20 (0.9)	0.03	25 (1.1)	0.15	33 (1.4)	0.01
**Hispanic**	40 (1.7)	33 (1.4)	-0.04	13 (0.6)	-0.04	38 (1.6)	-0.06
**Other**	19 (0.8)	25 (1.1)	0.10	26 (1.1)	0.07	34 (1.5)	0.02
**Unknown**	0 (0.0)	0 (0.0)	0.00	0 (0.0)	0.02	11 (0.5)	-0.02
**Trauma Center Proximity**							
**In county**	1,833 (78.8)	1,836 (78.9)	0.02	1,741 (74.8)	-0.14	1,818 (78.1)	-0.01
**Adjacent county**	399 (17.1)	411 (17.7)		461 (19.8)		413 (17.7)	
**No trauma center**	95 (4.1)	80 (3.4)		125 (5.4)		96 (4.1)	
**AIS Head/Neck ≥3**	0 (0.0)	0 (0.0)	N/A	0 (0.0)	N/A	0 (0.0)	N/A
**AIS Face ≥3**	0 (0.0)	0 (0.0)	N/A	0 (0.0)	N/A	0 (0.0)	N/A
**AIS Chest ≥3**	1,394 (59.9)	1,395 (59.9)	0.00	1,369 (58.8)	0.03	1,390 (59.7)	0.02
**AIS Abdomen/Pelvic ≥3**	0 (0.0)	0 (0.0)	N/A	0 (0.0)	N/A	0 (0.0)	N/A
**AIS Extremity ≥3**	0 (0.0)	0 (0.0)	N/A	0 (0.0)	N/A	0 (0.0)	N/A
**AIS External ≥3**	0 (0.0)	0 (0.0)	N/A	0 (0.0)	N/A	0 (0.0)	N/A

[a]SMD: Standardized mean difference, compared to Level I Trauma Center
[b]Other race includes beneficiaries reporting a race category not otherwise listed, include those reporting two or more races. Beneficiaries with unknown race are reported separately.

© 2022 Jarman MP et al. *JAMA Network Open.*

eTable 6: Descriptive statistics by trauma level, isolated traumatic brain injury

	Full Population						
**Traumatic Brain Injury**	**Level I**	**Level II**		**Level III**		**Non-Trauma**	
**Patient Characteristics**	**No. (%)**	**No. (%)**	**SMD[a]**	**No. (%)**	**SMD[a]**	**No. (%)**	**SMD[a]**
**Total (N)**	10,581	11,978		4,575		13,680	
**Age**							
**65-74**	2,588 (24.5)	2,597 (21.7)	-0.10	876 (19.1)	-0.19	2423 (17.7)	-0.22
**75-84**	4,037 (38.2)	4,619 (38.6)		1,775 (38.8)		4,990 (36.5)	
**≥85**	3,956 (37.4)	4,762 (39.8)		1,924 (42.1)		6,267 (45.8)	
**CCI**							
**0**	2,460 (23.2)	2,988 (24.9)	0.05	1,141 (24.9)	0.03	3,405 (24.9)	0.02
**1**	2,709 (25.6)	3,041 (25.4)		1,100 (24.0)		3,378 (24.7)	
**2**	1,917 (18.1)	2,064 (17.2)		839 (18.3)		2,383 (17.4)	
**≥3**	3,495 (33.0)	3,885 (32.4)		1,495 (32.7)		4,514 (33.0)	
**Frail**	5,642 (53.3)	6,736 (56.2)	-0.08	2,686 (58.7)	-0.15	8,305 (60.7)	-0.15
**Female**	5,139 (48.6)	6,191 (51.7)	-0.09	2,536 (55.4)	-0.19	7,528 (55.0)	-0.13
**Race**							
**White**	8,956 (84.6)	10,749 (89.7)	-0.22	4,138 (90.4)	-0.25	11,771 (86.0)	-0.04
**Black**	858 (8.1)	580 (4.8)	0.19	210 (4.6)	0.20	815 (6.0)	0.08
**Asian**	267 (2.5)	228 (1.9)	0.07	84 (1.8)	0.16	315 (2.3)	-0.03
**Hispanic**	296 (2.8)	242 (2.0)	0.03	54 (1.2)	-0.03	455 (3.3)	-0.05
**Other**	152 (1.4)	143 (1.2)	0.06	78 (1.7)	0.07	286 (2.1)	0.01
**Unknown**	52 (0.5)	36 (0.3)	0.04	11 (0.2)	0.06	38 (0.3)	0.03
**Trauma Center Proximity**							
**In county**	7,962 (75.2)	8,903 (74.3)	-0.04	3,381 (73.9)	-0.06	8,639 (63.2)	-0.26
**Adjacent county**	2,056 (19.4)	2,364 (19.7)		906 (19.8)		3,847 (28.1)	
**No trauma center**	563 (5.3)	711 (5.9)		288 (6.3)		1,194 (8.7)	
**AIS Head/Neck ≥3**	9,513 (89.9)	10,280 (85.8)	0.13	3,908 (85.4)	0.12	11,392 (83.3)	0.12
**AIS Face ≥3**	0 (0.0)	0 (0.0)	N/A	0 (0.0)	N/A	0 (0.0)	N/A
**AIS Chest ≥3**	0 (0.0)	0 (0.0)	N/A	0 (0.0)	N/A	0 (0.0)	N/A
**AIS Abdomen/Pelvic ≥3**	0 (0.0)	0 (0.0)	N/A	0 (0.0)	N/A	0 (0.0)	N/A
**AIS Extremity ≥3**	0 (0.0)	0 (0.0)	N/A	0 (0.0)	N/A	0 (0.0)	N/A
**AIS External ≥3**	0 (0.0)	0 (0.0)	N/A	0 (0.0)	N/A	0 (0.0)	N/A

© 2022 Jarman MP et al. *JAMA Network Open.*

	**After Propensity Score Matching**						
	**Level I**	**Level II**		**Level III**		**Non-Trauma**	
**Patient Characteristics**	**No. (%)**	**No. (%)**	**SMD[a]**	**No. (%)**	**SMD[a]**	**No. (%)**	**SMD[a]**
**Total (N)**	4,575	4,575		4,575		4,575	
**Age**							
**65-74**	875 (19.1)	875 (19.1)	0.00	876 (19.1)	0.00	875 (19.1)	0.00
**75-84**	1,777 (38.8)	1,777 (38.8)		1,775 (38.8)		1,777 (38.8)	
**≥85**	1,923 (42.0)	1,923 (42.0)		1,924 (42.1)		1,923 (42.0)	
**CCI**							
**0**	1,067 (23.3)	1,128 (24.7)	0.04	1,141 (24.9)	0.02	1,171 (25.6)	0.01
**1**	1,196 (26.1)	1,180 (25.8)		1,100 (24.0)		1,101 (24.1)	
**2**	822 (18.0)	785 (17.2)		839 (18.3)		793 (17.3)	
**≥3**	1,490 (32.6)	1,482 (32.4)		1,495 (32.7)		1,510 (33.0)	
**Frail**	2,495 (54.5)	2,540 (55.5)	-0.03	2,686 (58.7)	-0.12	2,751 (60.1)	-0.11
**Female**	2,262 (49.4)	2,370 (51.8)	-0.07	2,536 (55.4)	-0.17	2,469 (54.0)	-0.09
**Race**							
**White**	3,910 (85.5)	4,101 (89.6)	-0.18	4,138 (90.4)	-0.22	3,861 (84.4)	0.03
**Black**	355 (7.8)	242 (5.3)	0.14	210 (4.6)	0.19	309 (6.8)	0.04
**Asian**	105 (2.3)	93 (2.0)	0.09	84 (1.8)	0.16	106 (2.3)	-0.07
**Hispanic**	127 (2.8)	82 (1.8)	0.03	54 (1.2)	-0.05	183 (4.0)	-0.07
**Other**	57 (1.2)	46 (1.0)	0.03	78 (1.7)	0.05	99 (2.2)	0.00
**Unknown**	21 (0.5)	11 (0.2)	0.05	11 (0.2)	0.05	17 (0.4)	0.01
**Trauma Center Proximity**							
**In county**	3,418 (74.7)	3,417 (74.7)	0.00	3,381 (73.9)	-0.04	3,414 (74.6)	0.00
**Adjacent county**	899 (19.7)	904 (19.8)		906 (19.8)		908 (19.8)	
**No trauma center**	258 (5.6)	254 (5.6)		288 (6.3)		253 (5.5)	
**AIS Head/Neck ≥3**	3,949 (86.3)	3,951 (86.4)	0.00	3,908 (85.4)	0.02	3,949 (86.3)	0.00
**AIS Face ≥3**	0 (0.0)	0 (0.0)	N/A	0 (0.0)	N/A	0 (0.0)	N/A
**AIS Chest ≥3**	0 (0.0)	0 (0.0)	N/A	0 (0.0)	N/A	0 (0.0)	N/A
**AIS Abdomen/Pelvic ≥3**	0 (0.0)	0 (0.0)	N/A	0 (0.0)	N/A	0 (0.0)	N/A
**AIS Extremity ≥3**	0 (0.0)	0 (0.0)	N/A	0 (0.0)	N/A	0 (0.0)	N/A
**AIS External ≥3**	0 (0.0)	0 (0.0)	N/A	0 (0.0)	N/A	0 (0.0)	N/A

[a]SMD: Standardized mean difference, compared to Level I Trauma Center
[b]Other race includes beneficiaries reporting a race category not otherwise listed, include those reporting two or more races. Beneficiaries with unknown race are reported separately.

© 2022 Jarman MP et al. *JAMA Network Open.*

eTable 7: Descriptive statistics by trauma level, other isolated extremity fracture

	Full Population						
**Isolated Extremity Fracture**	**Level I**	**Level II**		**Level III**		**Non-Trauma**	
**Patient Characteristics**	**No. (%)**	**No. (%)**	**SMD[a]**	**No. (%)**	**SMD[a]**	**No. (%)**	**SMD[a]**
**Total (N)**	8,725	13,235		9,500		32,455	
**Age**							
**65-74**	2,812 (32.2)	3,939 (29.8)	-0.07	2,935 (30.9)	-0.01	8,913 (27.5)	-0.10
**75-84**	2,971 (34.1)	4,580 (34.6)		3,390 (35.7)		11,488 (35.4)	
**≥85**	2,942 (33.7)	4,716 (35.6)		3,175 (33.4)		12,054 (37.1)	
**CCI**							
**0**	2,911 (33.4)	4,388 (33.2)	0.08	3,031 (31.9)	0.02	10,818 (33.3)	0.05
**1**	1,987 (22.8)	3,257 (24.6)		2,326 (24.5)		7,994 (24.6)	
**2**	1,371 (15.7)	2,082 (15.7)		1,499 (15.8)		5,039 (15.5)	
**≥3**	2,456 (28.2)	3,508 (26.5)		2,644 (27.8)		8,604 (26.5)	
**Frail**	4,036 (46.3)	6,299 (47.6)	-0.04	4,608 (48.5)	-0.06	16,038 (49.4)	-0.06
**Female**	10,346 (78.2)	7,510 (79.1)	-0.07	25,964 (80.0)	-0.10	6,629 (75.9)	-0.10
**Race**							
**White**	12,260 (92.6)	8,840 (93.1)	-0.25	29,531 (90.9)	-0.27	7,631 (87.5)	-0.11
**Black**	577 (4.4)	355 (3.7)	0.23	1,598 (4.9)	0.27	721 (8.3)	0.13
**Asian**	132 (1.0)	107 (1.1)	0.09	368 (1.1)	0.12	118 (1.4)	0.02
**Hispanic**	96 (0.7)	52 (0.6)	0.01	381 (1.2)	-0.02	118 (1.4)	-0.03
**Other[b]**	136 (1.0)	117 (1.2)	0.05	473 (1.5)	0.03	96 (1.1)	0.02
**Unknown**	34 (0.3)	29 (0.3)	0.05	104 (0.3)	0.04	41 (0.5)	0.02
**Trauma Center Proximity**							
**In county**	9,936 (75.1)	7,103 (74.8)	-0.26	18,699 (57.6)	-0.28	7,201 (82.5)	-0.56
**Adjacent county**	2,647 (20.0)	1,898 (19.9)		10,496 (32.3)		1,278 (14.7)	
**No trauma center**	652 (4.9)	499 (5.3)		3,260 (10.0)		246 (2.8)	
**AIS Head/Neck ≥3**	0 (0.0)	0 (0.0)	N/A	0 (0.0)	N/A	0 (0.0)	N/A
**AIS Face ≥3**	0 (0.0)	0 (0.0)	N/A	0 (0.0)	N/A	0 (0.0)	N/A
**AIS Chest ≥3**	0 (0.0)	0 (0.0)	N/A	0 (0.0)	N/A	0 (0.0)	N/A
**AIS Abdomen/Pelvic ≥3**	0 (0.0)	0 (0.0)	N/A	0 (0.0)	N/A	0 (0.0)	N/A
**AIS Extremity ≥3**	2,557 (29.3)	3,700 (27.9)	0.02	2,800 (29.5)	-0.01	8,224 (25.3)	0.07
**AIS External ≥3**	0 (0.0)	0 (0.0)	N/A	0 (0.0)	N/A	0 (0.0)	N/A

© 2022 Jarman MP et al. *JAMA Network Open.*

	After Propensity Score Matching						
	Level I	Level II		Level III		Non-Trauma	
**Patient Characteristics**	**No. (%)**	**No. (%)**	**SMD[a]**	**No. (%)**	**SMD[a]**	**No. (%)**	**SMD[a]**
**Total (N)**	8,725	8,725		8,725		8,725	
**Age**							
**65-74**	2,812 (32.2)	2,807 (32.2)	0.00	2,800 (32.1)	0.00	2,811 (32.2)	0.00
**75-84**	2,971 (34.1)	2,975 (34.1)		2,986 (34.2)		2,973 (34.1)	
**≥85**	2,942 (33.7)	2,943 (33.7)		2,939 (33.7)		2,941 (33.7)	
**CCI**							
**0**	2,911 (33.4)	2,920 (33.5)	0.09	2,799 (32.1)	0.02	2,923 (33.5)	0.05
**1**	1,987 (2.8)	2,120 (24.3)		2,136 (24.5)		2,141 (24.5)	
**2**	1,371 (15.7)	1,400 (16.1)		1,378 (15.8)		1,347 (15.4)	
**≥3**	2,456 (28.2)	2,285 (26.2)		2,412 (27.6)		2,314 (26.5)	
**Frail**	4,102 (47.0)	4,212 (48.3)	-0.02	4,270 (48.9)	-0.06	4,036 (46.3)	-0.05
**Female**	6,629 (75.9)	6,786 (77.8)	-0.06	6,900 (79.1)	-0.11	6,929 (79.4)	-0.08
**Race**							
**White**	7,631 (87.5)	8,059 (92.4)	-0.23	8,115 (93.0)	-0.27	7,820 (89.6)	-0.07
**Black**	721 (8.3)	396 (4.5)	0.22	324 (3.7)	0.27	482 (5.5)	0.11
**Asian**	118 (1.4)	94 (1.1)	0.09	98 (1.1)	0.12	107 (1.2)	0.00
**Hispanic**	118 (1.4)	60 (0.7)	0.01	48 (0.6)	-0.02	118 (1.4)	-0.06
**Other[b]**	96 (1.1)	89 (1.0)	0.04	111 (1.3)	0.03	156 (1.8)	0.01
**Unknown**	41 (0.5)	27 (0.3)	0.04	29 (0.3)	0.03	42 (0.5)	0.00
**Trauma Center Proximity**							
**In county**	7,201 (82.5)	7,170 (82.2)	-0.01	6,902 (79.1)	-0.11	7,182 (82.3)	0.00
**Adjacent county**	1,278 (14.7)	1,310 (15.0)		1,547 (17.7)		1,297 (14.9)	
**No trauma center**	246 (2.8)	245 (2.8)		276 (3.2)		246 (2.8)	
**AIS Head/Neck ≥3**	0 (0.0)	0 (0.0)	N/A	0 (0.0)	N/A	0 (0.0)	N/A
**AIS Face ≥3**	0 (0.0)	0 (0.0)	N/A	0 (0.0)	N/A	0 (0.0)	N/A
**AIS Chest ≥3**	0 (0.0)	0 (0.0)	N/A	0 (0.0)	N/A	0 (0.0)	N/A
**AIS Abdomen/Pelvic ≥3**	0 (0.0)	0 (0.0)	N/A	0 (0.0)	N/A	0 (0.0)	N/A
**AIS Extremity ≥3**	2,557 (29.3)	2,543 (29.2)	0.00	2,555 (29.3)	-0.01	2,550 (29.2)	0.00
**AIS External ≥3**	0 (0.0)	0 (0.0)	N/A	0 (0.0)	N/A	0 (0.0)	N/A

[a]SMD: Standardized mean difference, compared to Level I Trauma Center
[b]Other race includes beneficiaries reporting a race category not otherwise listed, include those reporting two or more races. Beneficiaries with unknown race are reported separately.

© 2022 Jarman MP et al. *JAMA Network Open.*

eTable 8: Descriptive statistics by trauma level, other injuries

	Full Population						
**Other Injuries**	**Level I**	**Level II**		**Level III**		**Non-Trauma**	
**Patient Characteristics**	**No. (%)**	**No. (%)**	**SMD[a]**	**No. (%)**	**SMD[a]**	**No. (%)**	**SMD[a]**
**Total (N)**	7,970	10,825		6,575		24,106	
**Age**							
**65-74**	2,155 (27.0)	2,460 (22.7)	-0.14	1,500 (22.8)	-0.13	4,845 (20.1)	-0.18
**75-84**	2,740 (34.4)	3,832 (35.4)		2,332 (35.5)		8,357 (34.7)	
**≥85**	3,075 (38.6)	4,533 (41.9)		2,743 (41.7)		10,904 (45.2)	
**CCI**							
**0**	2,464 (30.9)	3,360 (31.0)	0.05	1,890 (28.7)	-0.01	7,359 (30.5)	0.03
**1**	1,855 (23.3)	2,558 (23.6)		1,595 (24.3)		5,846 (24.3)	
**2**	1,260 (15.8)	1,730 (16.0)		1,106 (16.8)		3,896 (16.2)	
**≥3**	2,391 (30.0)	3,177 (29.3)		1,984 (30.2)		7,005 (29.1)	
**Frail**	4,136 (51.9)	5,824 (53.8)	-0.05	3,570 (54.3)	-0.07	13,308 (55.2)	-0.07
**Female**	4,910 (61.6)	7,005 (64.7)	-0.09	4,386 (66.7)	-0.15	16,399 (68.0)	-0.13
**Race**							
**White**	6,958 (87.3)	9,997 (92.4)	-0.24	6,133 (93.3)	-0.29	21,863 (90.7)	-0.11
**Black**	576 (7.2)	405 (3.7)	0.22	230 (3.5)	0.23	1,078 (4.5)	0.12
**Asian**	136 (1.7)	131 (1.2)	0.09	67 (1.0)	0.16	296 (1.2)	0.01
**Hispanic**	169 (2.1)	137 (1.3)	0.04	53 (0.8)	0.02	467 (1.9)	-0.01
**Other**	106 (1.3)	113 (1.0)	0.06	78 (1.2)	0.08	349 (1.4)	0.04
**Unknown**	25 (0.3)	42 (0.4)	-0.02	14 (0.2)	0.03	53 (0.2)	0.02
**Trauma Center Proximity**							
**In county**	6,357 (79.8)	8,096 (74.8)	-0.16	4,915 (74.8)	-0.16	14,274 (59.2)	-0.45
**Adjacent county**	1,312 (16.5)	2,190 (20.2)		1,335 (20.3)		7,501 (31.1)	
**No trauma center**	301 (3.8)	539 (5.0)		325 (4.9)		2,331 (9.7)	
**AIS Head/Neck ≥3**	0 (0.0)	0 (0.0)	0.10	0 (0.0)	0.17	0 (0.0)	0.14
**AIS Face ≥3**	14 (0.2)	11 (0.1)	0.11	0 (0.0)	0.19	0 (0.0)	0.15
**AIS Chest ≥3**	314 (3.9)	315 (2.9)	-0.01	222 (3.4)	0.01	597 (2.5)	0.04
**AIS Abdomen/Pelvic ≥3**	235 (2.9)	264 (2.4)	-0.06	129 (2.0)	-0.11	297 (1.2)	-0.06
**AIS Extremity ≥3**	318 (4.0)	350 (3.2)	-0.04	206 (3.1)	-0.10	657 (2.7)	-0.07
**AIS External ≥3**	171 (2.1)	73 (0.7)	0.21	55 (0.8)	0.23	169 (0.7)	0.19

© 2022 Jarman MP et al. *JAMA Network Open.*

	After Propensity Score Matching						
	Level I	Level II		Level III		Non-Trauma	
**Patient Characteristics**	**No. (%)**	**No. (%)**	**SMD[a]**	**No. (%)**	**SMD[a]**	**No. (%)**	**SMD[a]**
**Total (N)**	6,575	6,575		6,575		6,575	
**Age**							
**65-74**	1,512 (23.0)	1,515 (23.0)	0.00	1,500 (22.8)	0.00	1,511 (23.0)	0.00
**75-84**	2,310 (35.1)	2,315 (35.2)		2,332 (35.5)		2,312 (35.2)	
**≥85**	2,753 (41.9)	2,745 (41.7)		2,743 (41.7)		2,752 (41.9)	
**CCI**							
**0**	2,014 (30.6)	2,033 (30.9)	0.05	1,890 (28.7)	0.00	2,019 (30.7)	0.05
**1**	1,515 (23.0)	1,572 (23.9)		1,595 (24.3)		1,634 (24.9)	
**2**	1,043 (15.9)	1,029 (15.7)		1,106 (16.8)		1,066 (16.2)	
**≥3**	2,003 (30.5)	1,941 (29.5)		1,984 (30.2)		1,856 (28.2)	
**Frail**	3,465 (52.7)	3,572 (54.3)	-0.05	3,570 (54.3)	-0.05	3,648 (55.5)	-0.06
**Female**	4,149 (63.1)	4,267 (64.9)	-0.05	4,386 (66.7)	-0.11	4,419 (67.2)	-0.09
**Race**							
**White**	5,778 (87.9)	6,075 (92.4)	-0.21	6,133 (93.3)	-0.26	5,891 (89.6)	-0.05
**Black**	442 (6.7)	239 (3.6)	0.20	230 (3.5)	0.21	317 (4.8)	0.08
**Asian**	109 (1.7)	81 (1.2)	0.08	67 (1.0)	0.16	81 (1.2)	-0.02
**Hispanic**	140 (2.1)	92 (1.4)	0.05	53 (0.8)	0.01	159 (2.4)	-0.03
**Other**	85 (1.3)	62 (0.9)	0.05	78 (1.2)	0.08	110 (1.7)	0.04
**Unknown**	21 (0.3)	26 (0.4)	-0.02	14 (0.2)	0.03	17 (0.3)	0.01
**Trauma Center Proximity**							
**In county**	5,243 (79.7)	5,244 (79.8)	0.00	4,915 (74.8)	-0.17	5,214 (79.3)	-0.01
**Adjacent county**	1,102 (16.8)	1,108 (16.9)		1,335 (20.3)		1,134 (17.2)	
**No trauma center**	230 (3.5)	223 (3.4)		325 (4.9)		227 (3.5)	
**AIS Head/Neck ≥3**	0 (0.0)	0 (0.0)	0.06	0 (0.0)	0.01	0 (0.0)	0.04
**AIS Face ≥3**	0 (0.0)	0 (0.0)	0.02	0 (0.0)	0.00	0 (0.0)	0.01
**AIS Chest ≥3**	218 (3.3)	192 (2.9)	0.02	222 (3.4)	-0.01	208 (3.2)	0.01
**AIS Abdomen/Pelvic ≥3**	164 (2.5)	146 (2.2)	0.01	129 (2.0)	-0.03	131 (2.0)	0.01
**AIS Extremity ≥3**	242 (3.7)	220 (3.3)	0.01	206 (3.1)	-0.03	232 (3.5)	0.01
**AIS External ≥3**	55 (0.8)	55 (0.8)	0.01	55 (0.8)	0.03	55 (0.8)	0.01

[a]SMD: Standardized mean difference, compared to Level I Trauma Center
[b]Other race includes beneficiaries reporting a race category not otherwise listed, include those reporting two or more races. Beneficiaries with unknown race are reported separately.

© 2022 Jarman MP et al. *JAMA Network Open.*

eTable 9: Descriptive statistics by trauma level, polytrauma

	Full Population						
**Polytrauma**	**Level I**	**Level II**		**Level III**		**Non-Trauma**	
**Patient Characteristics**	**No. (%)**	**No. (%)**	**SMD[a]**	**No. (%)**	**SMD[a]**	**No. (%)**	**SMD[a]**
**Total (N)**	10,830	10,382		4,122		10,229	
**Age**							
**65-74**	3,453 (31.9)	2,805 (27.0)	-0.17	1,046 (25.4)	-0.24	1717 (16.8)	-0.43
**75-84**	3,822 (35.3)	3,704 (35.7)		1,468 (35.6)		3,491 (34.1)	
**≥85**	3,555 (32.8)	3,873 (37.3)		1,608 (39.0)		5,021 (49.1)	
**CCI**							
**0**	3,932 (36.3)	3,788 (36.5)	0.01	1,374 (33.3)	-0.07	3,320 (32.5)	-0.07
**1**	2,763 (25.5)	2,604 (25.1)		1,050 (25.5)		2,563 (25.1)	
**2**	1,649 (15.2)	1,625 (15.7)		664 (16.1)		1,718 (16.8)	
**≥3**	2,486 (23.0)	2,365 (22.8)		1,034 (25.1)		2,628 (25.7)	
**Frail**	4,721 (43.6)	4,870 (46.9)	-0.09	1,980 (48.0)	-0.13	5,670 (55.4)	-0.24
**Female**	5,673 (52.4)	5,974 (57.5)	-0.15	2,509 (60.9)	-0.24	6,938 (67.8)	-0.32
**Race**							
**White**	9,685 (89.4)	9,640 (92.9)	-0.17	3,847 (93.3)	-0.20	9,451 (92.4)	-0.10
**Black**	555 (5.1)	282 (2.7)	0.18	107 (2.6)	0.19	279 (2.7)	0.12
**Asian**	204 (1.9)	144 (1.4)	0.05	68 (1.6)	0.16	139 (1.4)	0.02
**Hispanic**	223 (2.1)	164 (1.6)	0.00	31 (0.8)	-0.04	180 (1.8)	-0.03
**Other**	119 (1.1)	111 (1.1)	0.06	57 (1.4)	0.03	147 (1.4)	0.04
**Unknown**	44 (0.4)	41 (0.4)	0.00	12 (0.3)	0.03	33 (0.3)	0.01
**Trauma Center Proximity**							
**In county**	7,666 (70.8)	7,555 (72.8)	0.07	3,010 (73.0)	0.07	5,940 (58.1)	-0.26
**Adjacent county**	2,455 (22.7)	2,228 (21.5)		878 (21.3)		3,251 (31.8)	
**No trauma center**	709 (6.5)	599 (5.8)		234 (5.7)		1,038 (10.1)	
**AIS Head/Neck ≥3**	4,248 (39.2)	3,387 (32.6)	0.13	896 (21.7)	0.54	1,962 (19.2)	0.45
**AIS Face ≥3**	45 (0.4)	27 (0.3)	0.10	0 (0.0)	0.29	13 (0.1)	0.23
**AIS Chest ≥3**	3,749 (34.6)	2,618 (24.2)	0.26	975 (23.7)	0.28	1,362 (13.3)	0.42
**AIS Abdomen/Pelvic ≥3**	834 (7.7)	518 (5.0)	0.08	174 (4.2)	0.03	265 (2.6)	0.00
**AIS Extremity ≥3**	1,521 (14.0)	1,370 (13.2)	-0.03	620 (15.0)	-0.13	1,692 (16.5)	-0.14
**AIS External ≥3**	74 (0.7)	41 (0.4)	0.11	34 (0.8)	0.17	47 (0.5)	0.27

© 2022 Jarman MP et al. *JAMA Network Open.*

	After Propensity Score Matching						
	Level I	Level II		Level III		Non-Trauma	
Patient Characteristics	No. (%)	No. (%)	SMD[a]	No. (%)	SMD[a]	No. (%)	SMD[a]
**Total (N)**	4,122	4,122		4,122		4,122	
**Age**							
**65-74**	1,047 (25.4)	1,049 (25.4)	0.00	1,046 (25.4)	0.00	1,050 (25.5)	0.00
**75-84**	1,463 (35.5)	1,463 (35.5)		1,468 (35.6)		1,450 (35.2)	
**≥85**	1,612 (39.1)	1,610 (39.1)		1,608 (39.0)		1,622 (39.3)	
**CCI**							
**0**	1,503 (36.5)	1,519 (36.9)	0.01	1,374 (33.3)	-0.07	1,388 (33.7)	-0.06
**1**	1,053 (25.5)	1,009 (24.5)		1,050 (25.5)		1,028 (24.9)	
**2**	608 (14.8)	655 (15.9)		664 (16.1)		693 (16.8)	
**≥3**	958 (23.2)	939 (22.8)		1,034 (25.1)		1,013 (24.6)	
**Frail**	1,883 (45.7)	1,944 (47.2)	-0.04	1,980 (48.0)	-0.07	2,162 (52.5)	-0.14
**Female**	2,401 (58.2)	2,493 (60.5)	-0.06	2,509 (60.9)	-0.08	2,689 (65.2)	-0.14
**Race**							
**White**	3,708 (90.0)	3,839 (93.1)	-0.16	3,847 (93.3)	-0.17	3,753 (91.0)	-0.04
**Black**	190 (4.6)	107 (2.6)	0.15	107 (2.6)	0.15	134 (3.3)	0.07
**Asian**	73 (1.8)	59 (1.4)	0.06	68 (1.6)	0.16	68 (1.6)	0.02
**Hispanic**	87 (2.1)	63 (1.5)	0.02	31 (0.8)	-0.03	78 (1.9)	-0.05
**Other**	46 (1.1)	40 (1.0)	0.04	57 (1.4)	0.01	68 (1.6)	0.01
**Unknown**	18 (0.4)	14 (0.3)	0.02	12 (0.3)	0.03	21 (0.5)	-0.01
**Trauma Center Proximity**							
**In county**	3,260 (79.1)	3,010 (73.0)	0.09	3,018 (73.2)	-0.15	3,169 (76.9)	-0.08
**Adjacent county**	753 (18.3)	878 (21.3)		925 (22.4)		799 (19.4)	
**No trauma center**	109 (2.6)	234 (5.7)		179 (4.3)		154 (3.7)	
**AIS Head/Neck ≥3**	951(23.1)	939 (22.8)	0.01	896 (21.7)	0.01	883 (21.4)	0.03
**AIS Face ≥3**	0 (0.0)	0 (0.0)	0.02	0 (0.0)	-0.03	0 (0.0)	0.02
**AIS Chest ≥3**	1,022 (24.8)	989 (24.0)	0.02	975 (23.7)	0.01	801 (19.4)	0.08
**AIS Abdomen/Pelvic ≥3**	179 (4.3)	123 (3.0)	0.03	174 (4.2)	-0.02	98 (2.4)	0.04
**AIS Extremity ≥3**	571 (13.9)	536 (13.0)	0.01	620 (15.0)	-0.02	542 (13.1)	0.01
**AIS External ≥3**	34 (0.8)	32 (0.8)	0.01	34 (0.8)	0.02	33 (0.8)	0.06

[a]SMD: Standardized mean difference, compared to Level I Trauma Center
[b]Other race includes beneficiaries reporting a race category not otherwise listed, include those reporting two or more races. Beneficiaries with unknown race are reported separately.

© 2022 Jarman MP et al. *JAMA Network Open.*

eFigure 1: Standardized mean differences for matching variables, before and after propensity score matching

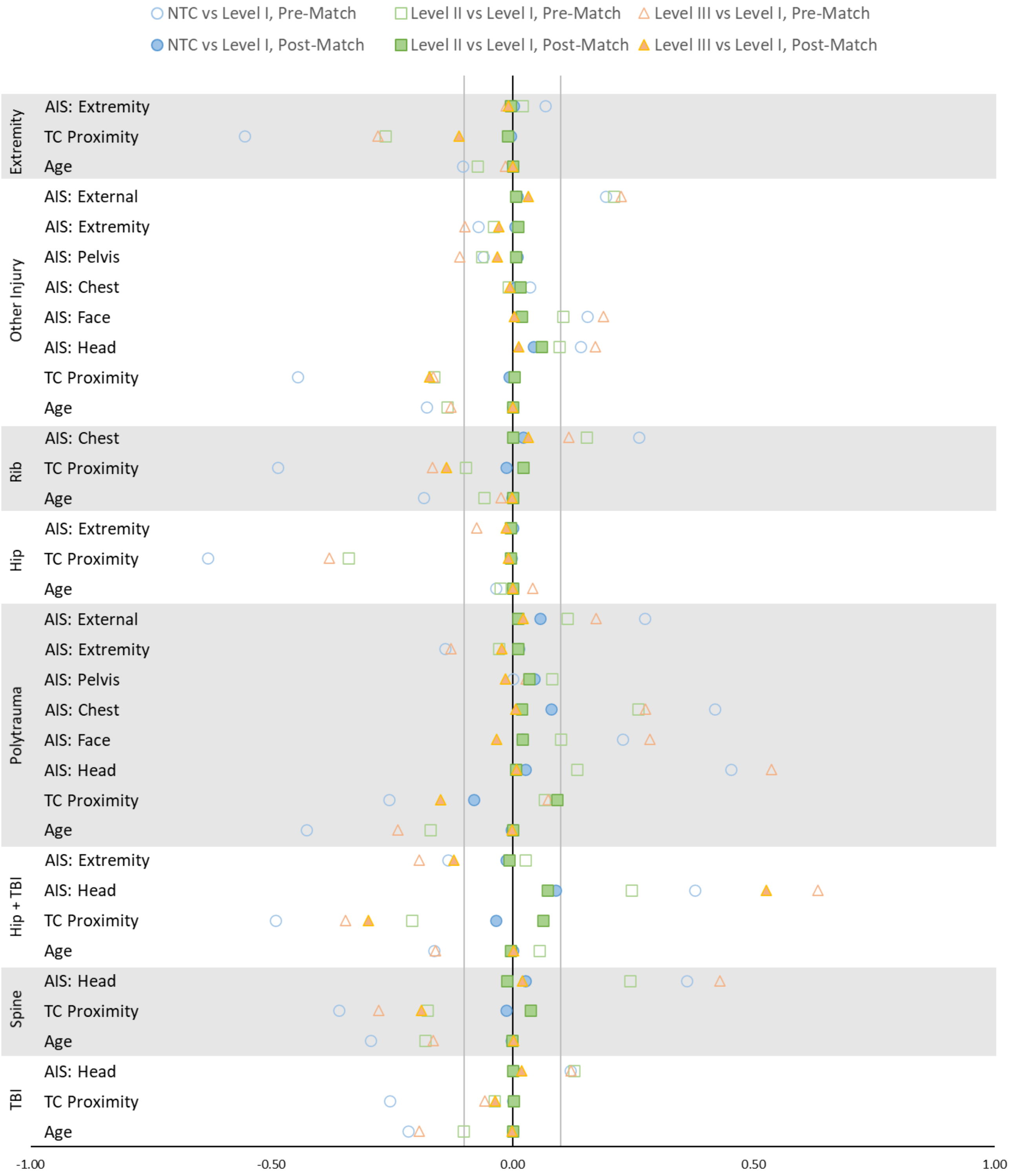

Pair-wise standardized mean differences for Level II, III and non-trauma centers compared to Level I, before and after propensity score matching.

© 2022 Jarman MP et al. *JAMA Network Open.*
